# A Novel CsYABBY3‐CsAS1 Feedback Loop Coordinates Trichome Differentiation and Cannabinoid Biosynthesis in *Cannabis sativa* L.

**DOI:** 10.1002/advs.75055

**Published:** 2026-04-02

**Authors:** Xuewen Zhu, Yaolei Mi, Xue Cao, Weiqiang Chen, Pucheng Fan, Jing Wang, Yiming Zhang, Wei Yang, Huihua Wan, Shanshan Chen, Xiangxiao Meng, Jun Li, Shuo Shen, Mingkun Huang, Xiaoyu Zhang, Ming Luo, Shilin Chen, Zhichao Xu, Wei Sun

**Affiliations:** ^1^ State Key Laboratory for Quality Ensurance and Sustainable Use of Dao‐di Herbs Institute of Chinese Materia Medica China Academy of Chinese Medical Sciences Beijing China; ^2^ Key Laboratory of National Forestry and Grassland Administration for Chinese Herbal Medicine College of Life Science Northeast Forestry University Harbin China; ^3^ Institute of Chinese Materia Medica and Artemisinin Research Center China Academy of Chinese Medical Sciences Beijing China; ^4^ Lushan Botanical Garden Chinese Academy of Sciences Jiujiang Jiangxi China; ^5^ College of Science National University of Defense Technology Changsha Hunan China; ^6^ South China Botanical Garden Chinese Academy of Sciences Guangzhou Guangdong China; ^7^ Institute of Herbgenomics Chengdu University of Traditional Chinese Medicine Chengdu China

**Keywords:** cannabinoid biosynthesis, feedback loop, FIL/YAB3‐AS1 transcriptional module, trichome differentiation

## Abstract

Glandular trichome development typically coincides with specialized‐metabolite production, yet the regulatory mechanisms that synchronizes these processes remain elusive. We uncover a trichome‐enriched CsYABBY3‐CsAS1 transcriptional module that links glandular trichome differentiation with cannabinoid biosynthesis in cannabis. CsYABBY3, a FILAMENTOUS FLOWER (FIL)/YAB3‐family transcription factor, promotes glandular trichome formation and elevates total cannabinoid levels. It directly binds a conserved TAATTAA motif in the promoters of *CsPT4* and *CsCBDAS*, activating their transcription. The R2R3‐MYB CsAS1 can also stimulate cannabinoid pathway gene expression independently, but its activity is enhanced in partnership with CsYABBY3. The two factors physically associate and reciprocally upregulate each other, forming a positive feedback loop that amplifies target‐gene output. A single residue (M199) in CsYABBY3 is required for CsAS1 interaction and cooperative promoter activation. Comparative evidence suggests broadly conserved FIL/YAB3‐AS1 coupling across species, alongside lineage‐specific differences in the molecular basis of complex formation. These findings define an engineerable regulatory circuit that connects trichome fate with specialized metabolism and provide a practical route to improve cannabinoid yield.

## Graphical abstract

A novel transcriptional module involving CsYABBY3 and CsAS1 is identified to regulate cannabinoid biosynthesis and trichome development. These factors mutually activate each other and form a protein complex via a conserved residue, acting synergistically to amplify metabolic flux through a coordinate feed‐forward mechanism.

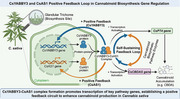

## 1 Introduction

*Cannabis sativa* L. is an herbaceous plant in the Cannabinaceae family, known for its medicinal and commercial value due to the production of cannabinoids, a class of terpenophenolic compounds [1]. These cannabinoids, which are primarily synthesized as acidic precursors such as Δ^9^‐tetrahydrocannabinolic acid (THCA) and cannabidiol acid (CBDA), are produced in the glandular trichomes of pistillate bracts and sugar leaves [2]. While Δ^9^‐tetrahydrocannabinol is known for psychoactive effects, cannabidiol has demonstrated therapeutic properties, such as in the treatment of refractory seizures [3]. The increasing global demand for cannabinoids has spurred significant interest in optimizing their production in *C. sativa*, making it critical to understand the regulatory mechanisms governing cannabinoid biosynthesis [4, 5].

Glandular trichomes are specialized epidermal structures essential for producing secondary metabolites, including cannabinoids. *Cannabis* trichomes, distributed on flowers, bracts, sugar leaves, and leaves, store phytocannabinoids, and their density correlates with cannabinoid content, making them an ideal system for studying trichome development and plant secondary metabolism [6, 7]. Cannabinoid biosynthesis originates from the enzymatic conversion of precursor compounds, olivetolic acid (OA) and geranyl diphosphate (GPP), into cannabigerolic acid (CBGA), which is subsequently converted into cannabidiolic acid (CBDA) and tetrahydrocannabinolic acid (THCA) by cannabinoid synthases CsCBDAS and CsTHCAS, respectively [8, 9, 10]. Emerging research has identified several transcription factors (TFs) that influence cannabinoid biosynthesis and trichome development. For instance, *CsMIXTA* and *CsMYC4* have been shown to regulate trichome initiation and activate genes involved in cannabinoid biosynthesis [11]. While individual factors controlling trichome density have been identified, the integrated regulatory modules that synchronize the morphological differentiation of glandular trichomes with the specialized metabolic pathways for cannabinoid biosynthesis are not yet understood.

Among the TFs involved in plant development, the *YABBY* gene family is notable for its role in lateral organ formation and trichome initiation [12, 13, 14]. YABBY genes, unique to seed plants, encode proteins defined by a C_2_C_2_ zinc‐finger domain and a YABBY‐specific domain [14, 15, 16, 17]. Phylogenetically, the family is classified into five clades: FILAMENTOUS FLOWER (FIL)/YAB3, YAB2, YAB5, CRABS CLAW (CRC), and INNER NO OUTER (INO) [18, 19]. The FIL/YAB3 subgroup is preferentially expressed in leaves and leaf‐derived organs and contributes to leaf polarity and trichome development [12, 18, 20, 21]. In *Arabidopsis thaliana*, YABBY proteins form homodimers or heterodimers with other TFs, such as AtKANADI, to modulate the development of lateral organs and trichome formation [22]. Emerging evidence further implicates YABBYs in glandular trichome development and the regulation of secondary metabolite accumulation; for example, *YABBY* genes have been linked to these processes in *Mentha spicata and Artemisia annua* [23, 24, 25].

In this study, we unveil a novel synergistic transcriptional module that coordinates trichome differentiation with cannabinoid biosynthesis. Departing from previously characterized single‐factor regulators, we demonstrated that the FIL/YAB3 member CsYABBY3 and the R2R3‐MYB protein CsAS1 establish a reciprocal positive feedback loop. Mechanistically, we identify a critical methionine residue (M199) in CsYABBY3 as indispensable for the physical interaction and functional synergy of this complex. These findings define a previously unknown CsAS1‐CsYABBY3‐*CsPT4*/*CsCBDAS* regulatory axis that couples trichome differentiation with specialized metabolism, providing a strategic route to orchestrate cannabinoid production in *C. sativa*.

## 2 Results

### 2.1 Identification and Characterization of the Cannabinoid Biosynthesis‐Related Transcription Factor *CsYABBY3*

**FIGURE 1:**
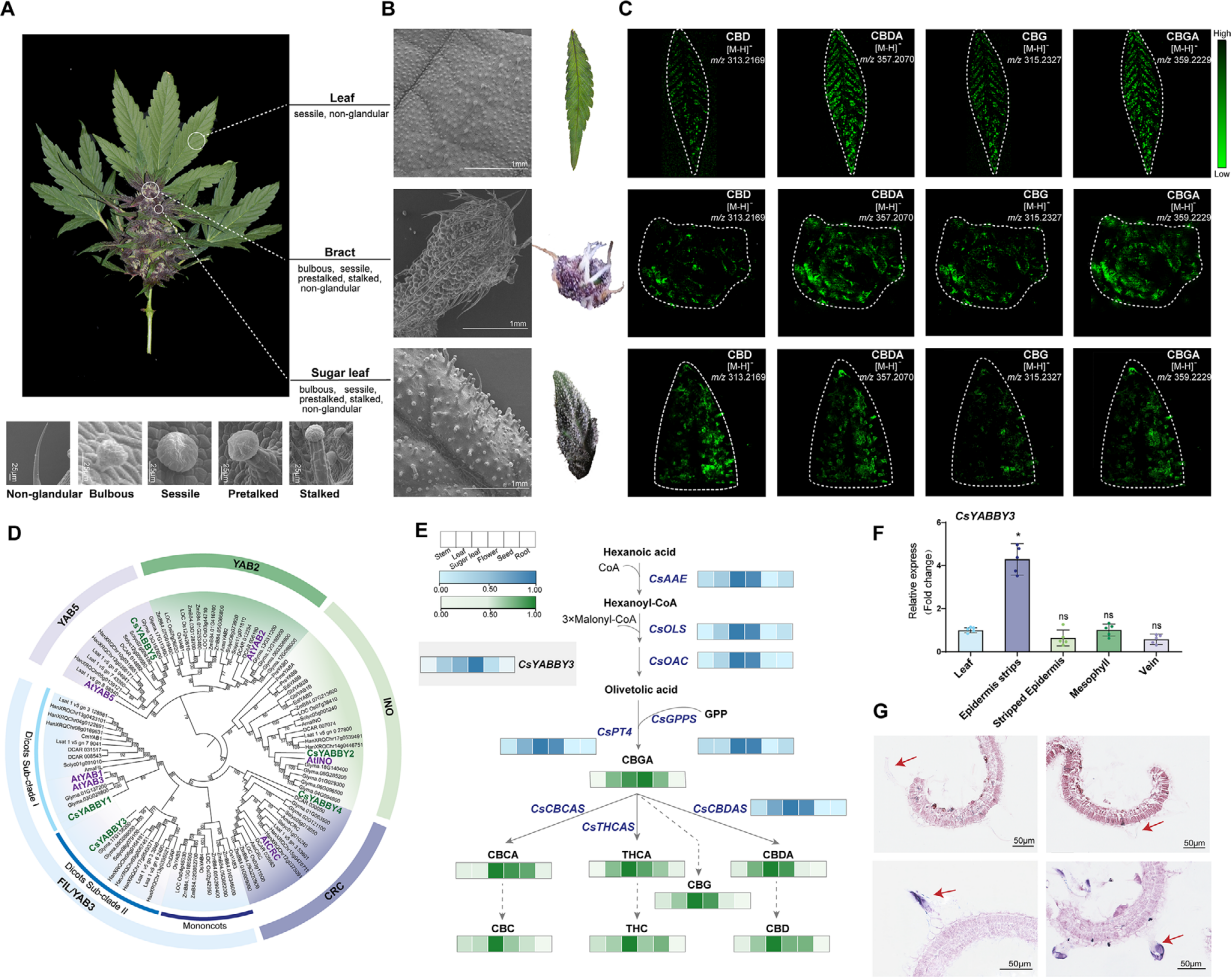
Identification and expression pattern of CsYABBY3 in *C. sativa*. (A) Scanning electron microscopy analysis illustrating the different GT types on the surface of various tissues in *C. sativa*. Bars = 25 µm. (B) Characteristic of trichomes of *C. sativa* leaves, bract, and sugar leaf using scanning electron microscope. Bars = 1 mm. (C) MALDI MSI images showing the relative distribution of major cannabinoids in leaves, bract, and sugar leaf of *C. sativa*, including CBD (m/z: 313.2173), CBDA (m/z: 357.2070), CBG (m/z: 315.2329), CBGA (m/z: 359.2228). All MALDI MSI images were obtained by extracting the ionized species at the M +e‐H adduct. (D) Phylogenetic tree of CsYABBYs and its homologs from other plant species. The maximum‐likelihood tree was constructed using the JTT model with 1000 bootstrap replicates. (E) Heatmap of enzymes and *CsYABBYs* (blue) along with cannabinoids (green) in the cannabinoid biosynthesis pathway of the Cannabis cultivar ‘DiKu’ (DiKu: Dinamed Kush). Data of the transcriptional level was present by log_2_ (FPKM + 1) and represented on a color scale (high: blue or green; low: white). (F) qPCR analysis of the expression level of *CsYABBY3* in distinct leaf tissues. The statistical analysis was performed with one‐way ANOVA. * indicates *p* < 0.05 and ns represents not significant. (G) In situ hybridization of *CsYABBY3* in vegetative leaf sections. Arrows indicate glandular trichomes (with storage cavity) and non‐glandular trichome. The negative control (above panel) was performed with a sense probe. Scale bar = 50 µm.

In *C. sativa*, glandular trichomes are primarily localized on the pistillate bract surfaces and associated bracteal tissues (Figure 1A), functioning as specialized metabolic factories for cannabinoid biosynthesis and storage [26]. To establish the spatial coordination between trichome distribution and cannabinoid deposition, we performed high‐resolution matrix‐assisted laser desorption/ionization mass spectrometry imaging (MALDI‐MSI), revealing an obvious accumulation of cannabidiol (CBD), cannabigerol (CBG), CBDA, and CBGA predominantly within glandular trichomes (Figure 1B,C; Figures S1A).

To identify the regulators of cannabinoid biosynthesis, we conducted a weighted gene co‐expression network analysis (WGCNA) using transcriptomes from six organs of *C. sativa* (cv. DiKu): flower, bracts, sugar leaf, leaf, stem, seed, and root. This analysis recovered a module enriched with cannabinoid biosynthetic genes, including *CsPT4*, *CsCBDAS*, and *CsGPPS*, within which 72 TFs co‐clustered. To ensure the identified regulators are functionally conserved across different genetic backgrounds, we integrated our organ‐level transcriptome data (Group A) with a publicly released trichome‐specific dataset (Group B), covering a total of eight distinct cultivars. By applying a threshold of FPKM > 10, we identified 12 candidate TFs that consistently exhibited both flower/sugar leaves‐specific and trichome enriched expression across all sampled cultivars (Figure S1B,C). Notably, a member of the YABBY family, designated as *CsYABBY3*, was annotated.

A genome‐wide survey identified five *YABBY* genes in *C. sativa*, and phylogenetic analysis indicated that the co‐expressed gene belongs to the FIL/YAB3 clade (Figure 1D; Table S1). The expression pattern of *CsYABBY3* correlated with cannabinoid accumulation, particularly total CBD and CBG (Figure 1E; Figure S1D). Given the expression pattern, qRT‐PCR validation showed that only *CsYABBY3* was significantly expressed in the trichome‐rich epidermal strips of leaves (Figure 1F; Figure S1E). Domain analysis revealed that CsYABBY3 encodes a 235‐amino acid protein with two conserved domains: an N‐terminal C_2_C_2_ zinc finger domain and a C‐terminal YAB domain (Figure S2). Furthermore, we performed RNA in situ hybridization to determine the spatial expression of CsYABBY3 and found that it was predominantly localized to the trichomes in *C. sativa* (Figure 1G). These findings suggest a potential regulatory role for CsYABBY3, a member of the FIL/YAB3 subgroup, in the biosynthesis of cannabinoids within cannabis trichomes.

### 2.2 CsYABBY3 Coordinated Trichome Differentiation and Cannabinoid Biosynthesis

**FIGURE 2:**
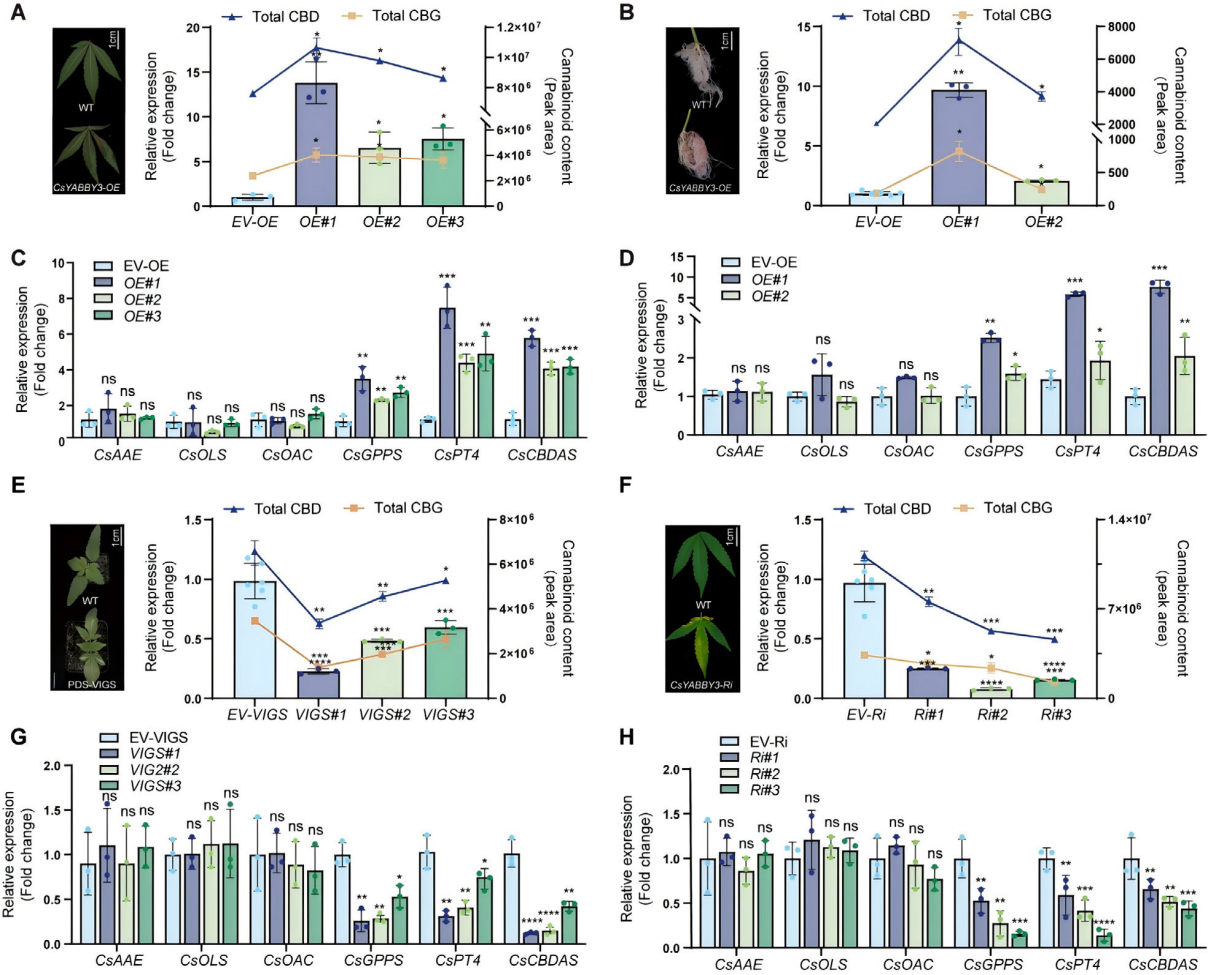
Regulation of cannabinoid content and expression of CBDA biosynthetic genes by CsYABBY3 in *C. sativa. (*A, B) Total CBD and CBG contents in transient *CsYABBY3‐*OE cannabis leaves (A) and stable *CsYABBY3‐OE* hairy roots (B). (C, D) qRT‐PCR analysis of CBDA biosynthetic genes in transient *CsYABBY3‐*OE cannabis leaves (C) and stable *CsYABBY3‐*OE hairy roots (D). (E, F) Total CBD and CBG contents in *CsYABBY3*‐silenced lines mediated by VIGS (E) and RNAi (F). (G, H) qRT‐PCR analysis of CBDA biosynthetic genes in *CsYABBY3*‐silenced lines mediated by VIGS (G) and RNAi (H). Transient expression assays were performed by *Agrobacterium*‐mediated vacuum infiltration. Scale Bar: 1 cm. Data represent at least three independent replicates.

To elucidate the functional role of CsYABBY3 in CBDA biosynthesis, we transiently overexpressed this TF in *C. sativa* leaves (Figure 2A) and generated stable transgenic hairy root lines (Figure 2B), utilizing RUBY reporter to validate overexpression. LC‐MS quantification revealed significantly higher total CBD and CBG accumulation in *CsYABBY3‐OE* leaves and hairy roots than in controls (Figure 2A,B). Consistently, *CsYABBY3‐OE* in both the transient assay (Figure 2C) and stable hairy root system (Figure 2D) upregulated key cannabinoid biosynthetic genes, including *CsGPPS*, *CsPT4*, and *CsCBDAS*.

We further employed three loss‐of‐function approaches, including virus‐induced gene silencing (VIGS), RNA interference (RNAi), and antisense oligonucleotide (AsODN). Relative to the TRV2 control group, TRV*‐CsYABBY3* lines exhibited significantly reduced *CsYABBY3* transcript abundance and, correspondingly, lower total CBD and CBG (Figure 2E). Consistent with the VIGS results, *CsYABBY3* suppression by RNAi and AsODN similarly decreased cannabinoid accumulation (Figure 2F; Figure S3B). qRT‐PCR further revealed marked downregulation of *CsGPPS*, *CsPT4*, and *CsCBDAS* in TRV2‐*CsYABBY3*, *CsYABBY3*‐RNAi, and *CsYABBY3*‐AsODN lines compared with their respective controls (Figure 2G,H; Figure S3C). Collectively, these results demonstrate that CsYABBY3 functions as a transcriptional activator that orchestrates pathway gene expression to modulate cannabinoid biosynthesis in *C. sativa*.

To test whether CsYABBY3 regulates trichome development, we generated *CsYABBY3‐OE* tomato plants. Scanning electron microscopy (SEM) revealed that the densities of both glandular and non‐glandular trichomes were significantly increased in *CsYABBY3‐OE* plants relative to wild type (WT) (Figure S4A–E). In two independent *CsYABBY3*‐*OE* lines, trichome density increased by 2.5‐fold and 6.5‐fold compared with WT. Given that cannabinoids and monoterpenes share the 2‐C‐methyl‐D‐erythritol 4‐phosphate (MEP) pathway, we profiled terpenoid metabolites in leaves and fruits of *CsYABBY3‐OE* tomatoes plants. Compared with WT, *CsYABBY3*‐*OE* lines accumulated significantly more total terpenoids in both organs and showed increased GPP in fruits (Figure S4F,G). Together, these findings support a role for CsYABBY3 in regulating trichome development and terpenoid biosynthesis, potentially through modulation of the MEP pathway.

### 2.3 CsYABBY3 Activated the Expression of *CsPT4* and *CsCBDAS* in *C. sativa* by Directly Binding to Their Promoters

**FIGURE 3:**
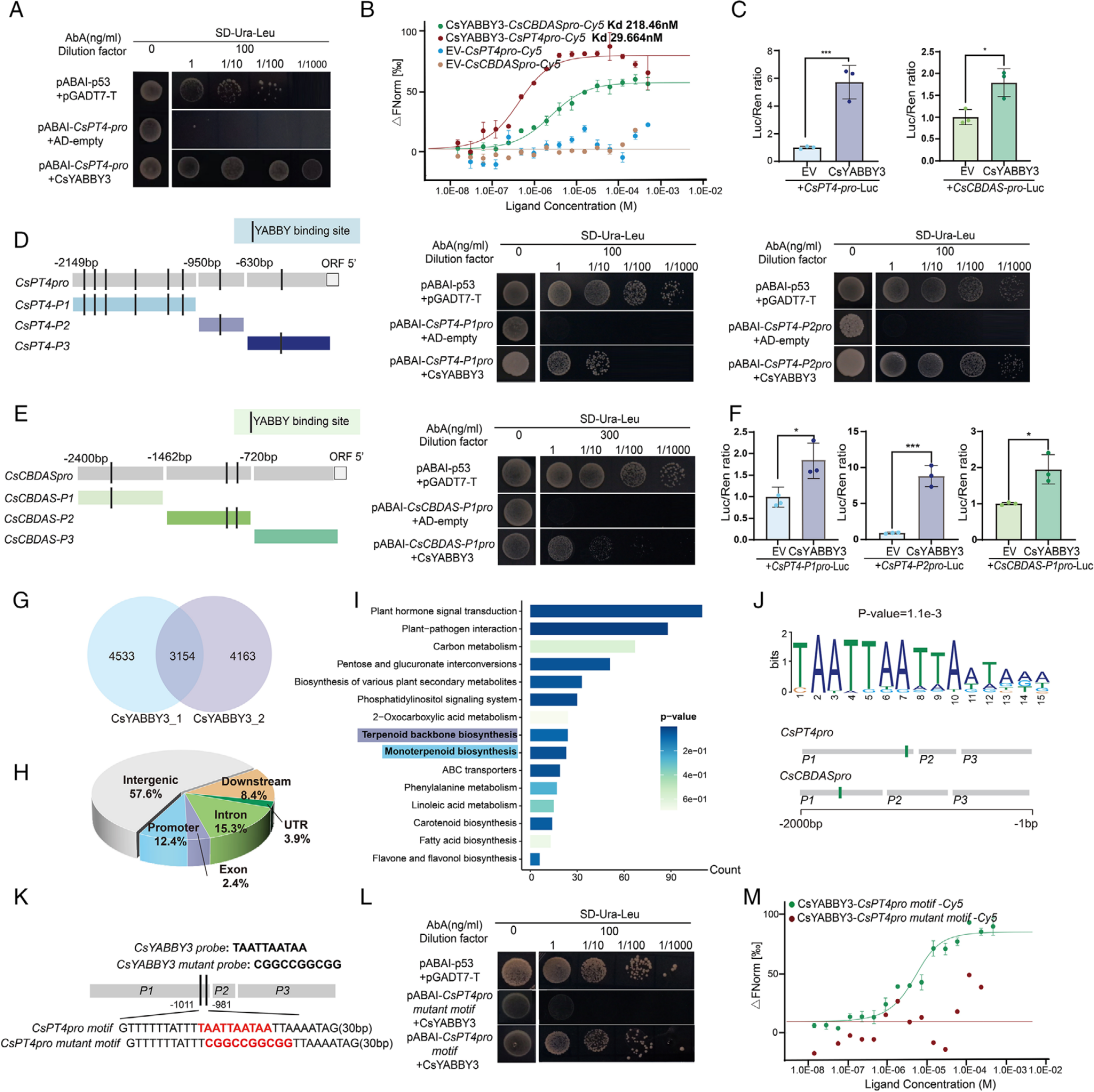
CsYABBY3 directly activates the expression of *CsPT4* and *CsCBDAS*. (a) Y1H assay showing CsYABBY3 binding to *CsPT4pro*. (b) MST analysis of CsYABBY3 interactions with Cy5‐labeled *CsPT4pro* and *CsCBDASpro*. (c) Dual‐LUC assay in *N. benthamiana* leaves demonstrating transcriptional activation of *CsPT4pro* and *CsCBDASpro* by CsYABBY3. (d, e) Truncated promoter analyses of *CsPT4pro* (P1‐P3) and *CsCBDASpro* (P1‐P3) tested via Y1H for CsYABBY3 binding. (f) Dual‐LUC assay showing that CsYABBY3 activates *CsPT4‐P1pro*, *CsPT4‐P2pro*, and *CsCBDAS‐P1pro* in *N. benthamiana* leaves. (g) Overlapping binding peaks (3154) identified from the two biological replicates of DAP‐seq. (h) Genomic distribution of CsYABBY3 binding sites relative to gene features. (i) KEGG annotations of genes nearest to the 3154 DAP‐seq peaks, highlighting the Terpenoid backbone biosynthesis and Monoterpene biosynthesis pathway. (j) The highest‐scoring DNA motif identified from CsYABBY3 DAP‐seq peaks and its mapping to the *CsPT4pro* and *CsCBDASpro*. (k) Wild‐type (WT) and mutant probes used in Y1H and MST. Red letters represent the core motif bound by CsYABBY3, mutated to ‘CGGCCGGCGG’ in the assay. (l) Y1H confirming CsYABBY3 binding to the *CsPT4pro* motif. (m) MST validating the interaction between *CsPT4pro* and CsYABBY3. Data represent at least three biological replicates.

Subcellular localization showed that CsYABBY3 was localized in the nucleus (Figure S5). To clarify the regulatory role of CsYABBY3, we performed Y1H assays, which demonstrated that CsYABBY3 directly bound to the full‐length promoters of *CsPT4* (*CsPT4pro*) (Figure 3A). The full‐length promoters of *CsCBDAS* (*CsCBDASpro*) exhibited strong self‐activation (Figure S6A). MST analysis further confirmed the direct binding of CsYABBY3 to both *CsPT4* and *CsCBDAS* promoters, with binding affinity (Kd) of 29.66 and 218.46 nm, respectively (Figure 3B). To assess the functional impact of CsYABBY3 binding to these promoters, we conducted Dual‐LUC assays. Co‐expression of *35S:CsYABBY3* and *CsPT4pro:LUC* or *CsCBDASpro:LUC* reporter constructs resulted in significantly higher reporter gene expression in *N. benthamiana* (Figure 3C), suggesting that CsYABBY3 activates the transcription of *CsPT4* and *CsCBDAS*. Collectively, these findings establish CsYABBY3 as a nuclear‐localized transcriptional activator that directly targets *CsPT4* and *CsCBDAS* promoters to drive CBDA biosynthesis.

To investigate the *cis*‐regulatory elements recognized by CsYABBY3 in the ∼2 kb promoter regions upstream of *CsPT4* and *CsCBDAS*, we performed computational predictions of TF binding sites using the JASPAR database. The analysis identified seven potential CsYABBY3‐binding motifs in the *CsPT4pro* and three in the *CsCBDASpro* (Figure 3D,E). Based on the motif distribution, we generated three sequential promoter truncation constructs for both *CsPT4pro* and *CsCBDASpro* (designated as *P1‐P3pro*), none of which exhibited transcriptional self‐activation (Figure S6B–D). Y1H and Dual‐LUC assays demonstrated specific binding interactions between CsYABBY3 and the *CsPT4‐P1pro*, *CsPT4‐P2pro*, and *CsCBDAS‐P1pro* fragments (Figure 3D‐F, Supporting Information).

To delineate the precise binding loci of CsYABBY3, we performed DAP‐seq, achieving a 99% mapping rate of clean reads to the reference genome and identifying 3154 CsYABBY3‐binding peaks across all 10 chromosomes (Figure 3G; Figure S7A). Approximately 12.4% of the binding peaks were located within 2 kb upstream of the transcription start site, corresponding to the promoter region (Figure 3H; Figure S7B). KEGG pathway and Gene Ontology (GO) enrichment analyses of binding peak‐associated target genes identified enriched terms related to Terpenoid backbone biosynthesis and Monoterpenoid biosynthesis (Figure 3I), as well as fatty acid metabolic process (Figure S7C). The highest‐scoring *cis*‐acting element from DAP‐seq data, TAATTAA, was found in both *CsPT4pro* and *CsCBDASpro*, indicating that these promoters are direct targets of CsYABBY3 (Figure 3J,K). The sequence‐specific interaction between CsYABBY3 and the core motif (−1011∼−981 bp) in *CsPT4‐P1pro* was further validated by independent Y1H assays (Figure 3L) and MST analysis (Figure 3M). Importantly, mutation of the TAATTAA motif to CGGCCGGCGG completely abolished the binding in both assays, providing definitive evidence for the motif‐specific recognition of CsYABBY3.

### 2.4 CsAS1 Binds to the M199 Residue of the CsYABBY3 Protein

**FIGURE 4:**
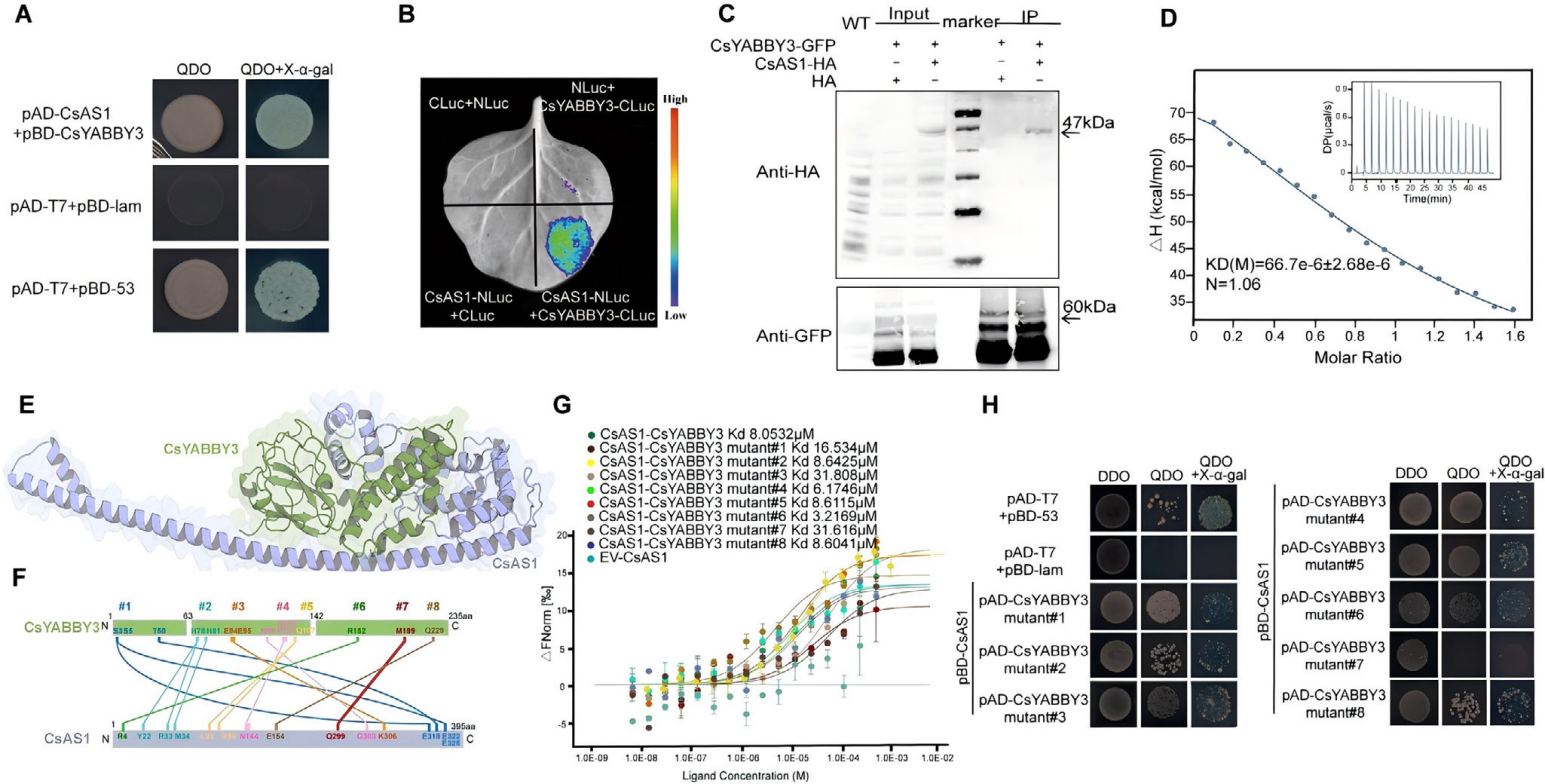
Interaction mechanism between CsYABBY3 and CsAS1. (A, B) Y2H assay (A) and LCI assay (B) showing the protein‐protein interaction between CsYABBY3 and CsAS1. (C) Co‐IP assay confirming CsYABBY3‐CsAS1 interaction. GFP‐tagged CsYABBY3 and HA‐tagged CsAS1 were co‐expressed in *N. benthamiana* leaves. Total protein extracts were immunoprecipitated using an anti‐GFP antibody. The arrow at ∼47 kDa indicates CsAS1‐HA, and the arrow at ∼60 kDa indicates CsYABBY3‐GFP. WT, wild type. (D) ITC analysis of the CsYABBY3‐CsAS1 interaction. (E) Predicated protein structure and molecular docking of CsYABBY3 and CsAS1. (F) Schematic diagram of the CsYABBY3‐CsAS1 interface highlighting eight interaction sites (#1‐#8). (G) MST assays assessing the interaction between CsAS1 and CsYABBY3. (H) Y2H assays verifying interactions between CsYABBY3 mutant#1‐#8 and CsAS1.

To identify potential interacting protein partners of CsYABBY3 implicated in the regulation of cannabinoid biosynthesis, we performed yeast two‐hybrid (Y2H) library screening (Table S2). Among the candidates, a trichome‐enriched R2R3‐MYB transcription factor was identified and designated CsAS1 (ASYMMETRIC LEAF1). This interaction was functionally validated through Y2H (Figure 4A) and firefly luciferase complementation imaging (LCI) (Figure 4B). Co‐immunoprecipitation (Co‐IP) experiments further corroborated the interaction between CsAS1‐HA and CsYABBY3‐GFP fusion proteins (Figure 4C). Isothermal Titration Calorimetry (ITC) analysis revealed a Kd of 4.74 ± 0.56 µm with a 1:1 binding stoichiometry (N = 1.06), indicating moderate binding affinity (Figure 4D). Subcellular localization showed nuclear enrichment of CsAS1, consistent with a role as a transcriptional regulator (Figure S8A).

To elucidate the molecular basis of the CsYABBY3‐CsAS1 interaction, we divided the CsYABBY3 coding sequence into three distinct fragments based on the conserved YABBY protein domains: an N‐terminal zinc finger domain (P1), a middle region lacking conserved motifs (P2), and a C‐terminal YABBY domain (P3) (Figure S8A,B). Transcriptional auto‐activation assays confirmed that CsAS1 had no self‐activation activity. Y2H assays revealed a direct interaction between CsAS1 and the C‐terminal YABBY domain (CsYABBY3‐P3). Molecular docking predicted a high‐affinity heterodimeric complex (Figure 4E) with eight putative hydrogen‐bonding sites (#1‐#8) spanning the protein‐protein interface, with bond lengths ranging from 1.5 to 4.2 Å (Figure 4F; Figure S8C).

MST analysis quantified the binding affinities of site‐directed CsYABBY3 interface mutants (#1‐#8; Figure S8) to CsAS1, yielding Kd of 3.22–31.62 µm, compared with 8.05 µm for the wild‐type CsYABBY‐CsAS1 complex (Figure 4G). Among these, mutant #7 exhibited significantly reduced affinity. Y2H assays confirmed that only the CsYABBY3 mutant #7 failed to grow on QDO medium supplemented with X‐α‐Gal (Figure 4H), highlighting residue M199 (site #7) as indispensable for the CsYABBY3‐CsAS1 interaction in vivo.

### 2.5 CsAS1 Is a Positive Regulator of CBDA Biosynthesis

Organs‐specific expression profiling demonstrated strong co‐expression of *CsAS1* with *CsYABBY3*, *CsPT4*, and *CsCBDAS* across cannabinoid‐rich organs of different cultivars (Figure S1B). Subcellular localization showed CsAS1 was located in nucleus (Figure S9A). RNA in situ hybridization localized *CsAS1* transcripts exclusively to glandular trichomes, mirroring the expression pattern of *CsYABBY3* (Figure S9B).

**FIGURE 5:**
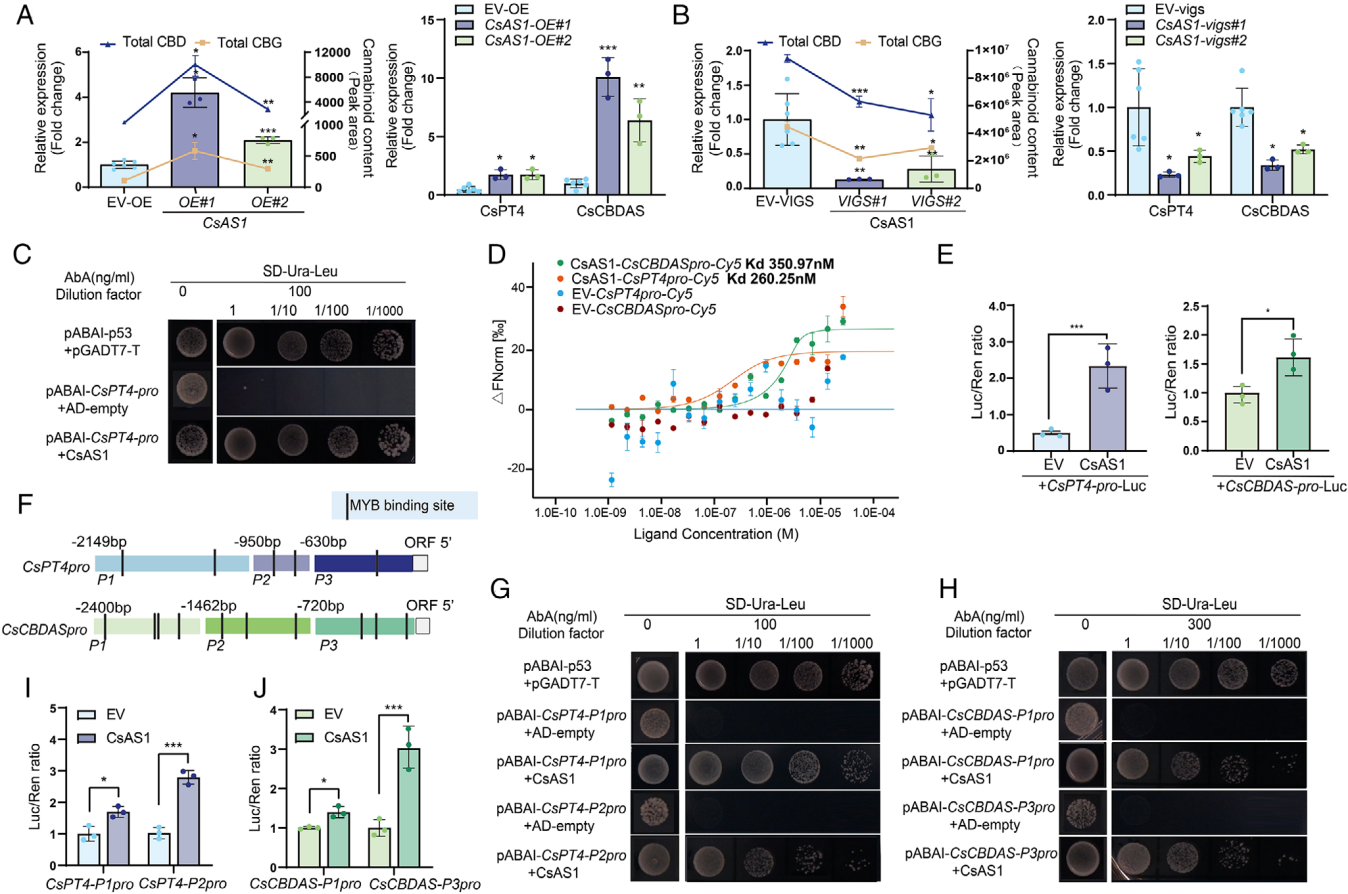
CsAS1 is a positive regulator of CBDA biosynthesis. (a) The total CBD and CBG (left) and the expression of *CsPT4* and *CsCBDAS* (right) in *CsAS1*‐OE stably transformed hairy roots. (b) The total CBD and CBG (left), and the expression of *CsPT4* and *CsCBDAS* (right) in VIGS‐mediated *CsAS1‐*silenced lines. (c) Y1H assay showing CsAS1 binding to *CsPT4pro*. (d) MST analysis of CsAS1 interactions with Cy5‐labeled *CsPT4pro* and *CsCBDASpro*. (e) Dual‐LUC assay showing that CsAS1 activates *CsPT4pro* and *CsCBDASpro* in *N. benthamiana* leaves. (f) The potential binding sites of CsAS1 on *CsPT4pro* and *CsCBDASpro* predicted by JASPAR. (g, h) Y1H assays showing that CsAS1 binding to *CsPT4‐P1pro* and *CsPT4‐P2pro* (G), as well as *CsCBDAS‐P1pro* and *CsCBDAS‐P3pro* (h). (i, j) Dual‐Luc assay showing that CsAS1 activates *CsPT4‐P1pro* and *CsPT4‐P2pro* (I), as well as *CsCBDAS‐P1pro* and *CsCBDAS‐P3pro* (j) in *N. benthamiana* leaves. Data represent at least three biological replicates.

To investigate CsAS1 function, we applied the same framework used for CsYABBY3. *CsAS1*‐OE lines in transient cannabis leaves (Figure S10A) and hairy root transformation systems (Figure 5A) revealed a significant increase in total CBD and CBG accumulation relative to controls. Correspondingly, transcriptional suppression of *CsAS1* by VIGS and AsODN reduced *CsAS1* expression and markedly decreased total CBD and CBG (Figure 5B; Figure S10B). Parallel qRT‐PCR analyses demonstrated substantial downregulation of *CsPT4* and *CsCBDAS* in silenced lines (Figure 5A,B, right panels), supporting CsAS1 as a transcriptional activator of these key cannabinoid biosynthetic genes in *C. sativa*.

Y1H assays demonstrated direct binding of CsAS1 to *CsPT4pro* (Figure 5C). MST further quantified this interaction, revealing high‐affinity binding of CsAS1 to *CsPT4pro* (Kd = 260.25 nm) and *CsCBDAS* (Kd = 350.97 nm) (Figure 5D). Dual‐LUC assays showed that co‐expression of *35S:CsAS1* with *CsPT4pro:LUC* or *CsCBDASpro:LUC* significantly enhanced reporter activity (Figure 5E), confirming CsAS1 as a transcriptional activator. We predicted at least one putative CsAS1‐binding motif in each truncated fragment of *CsPT4pro* and *CsCBDASpro* (Figure 5F). Promoter truncation assays indicated that CsAS1 directly bound to *CsPT4‐P1pro*, *CsPT4‐P2pro*, *CsCBDAS‐P1pro*, and *CsCBDAS‐P3pro* fragments (Figure 5G‐J). Together, these results demonstrate that CsAS1 directly regulates cannabinoid biosynthetic genes at the transcriptional level in *C. sativa*.

### 2.6 CsYABBY3 and CsAS1 Established a Positive Feedback Loop and Synergistically Regulate CBDA Biosynthesis in *C. sativa*

**FIGURE 6:**
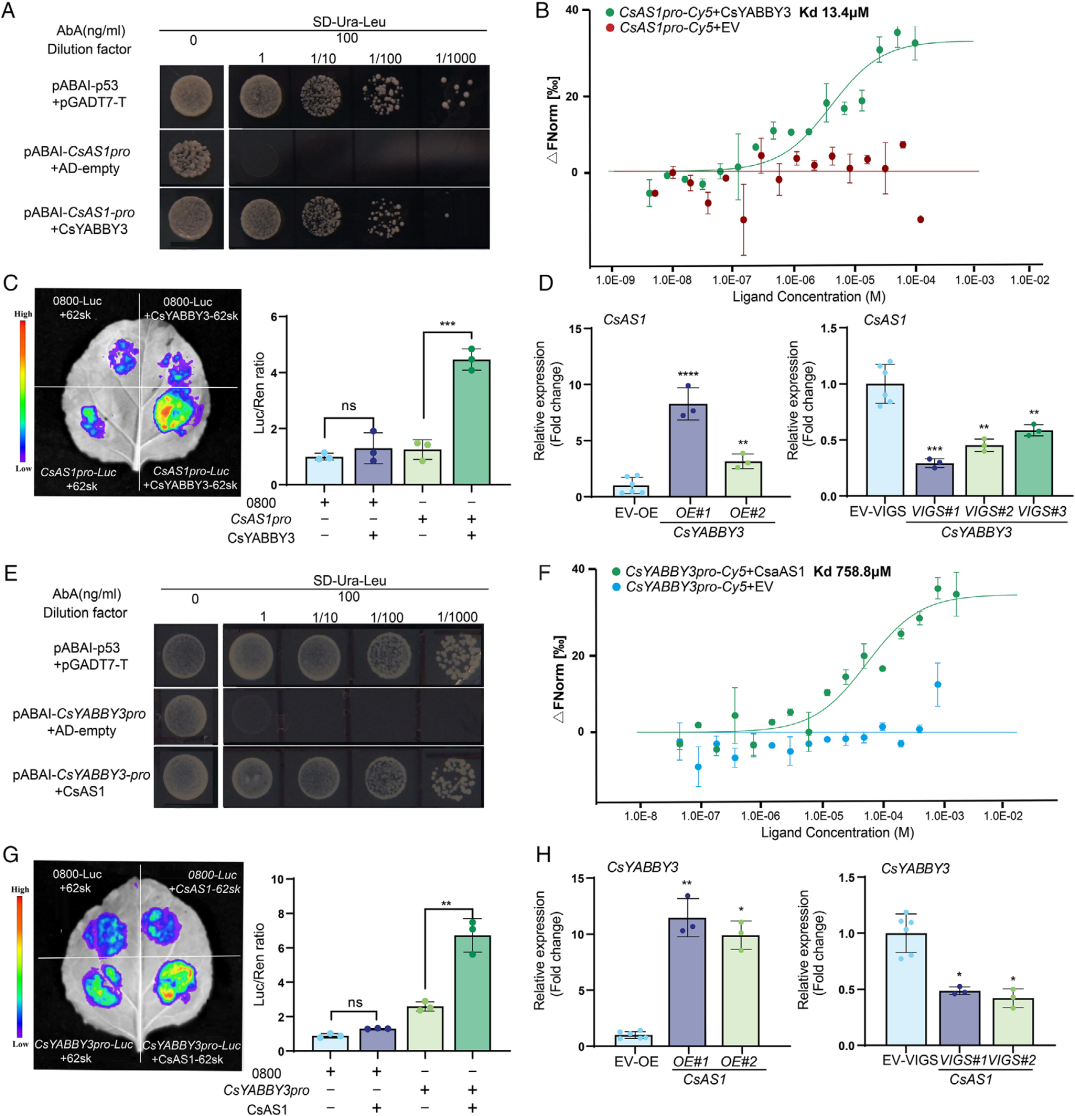
Mutual activation between CsYABBY3 and CsAS1 establishes a positive feedback loop. (a) Y1H assay showing CsYABBY3 binding to *CsAS1pro*. (b) MST analysis of the interaction between Cy5‐labeled *CsAS1pro* (target) and CsYABBY3 protein (ligand). (c) Dual‐LUC assay demonstrating the activation of *CsAS1pro* by CsYABBY3 in *N. benthamiana* leaves. (d) qRT‐PCR analysis of *CsAS1* expression in *CsYABBY3*‐OE and VIGS‐mediated CsYABBY3‐silenced lines. (e) Y1H assay showing CsAS1 binding to *CsYABBY3pro*. (f) MST analysis of the interaction between Cy5‐labeled *CsYABBY3pro* (target) and CsAS1 protein (ligand). (g) Dual‐LUC assay demonstrating the activation of *CsYABBY3pro* by CsAS1 in *N. benthamiana* leaves. (h) qRT‐PCR analysis of *CsYABBY3* expression in *CsAS1*‐OE and *CsAS1*‐silenced lines. Data represent at least three biological replicates.

To define the regulatory relationship between CsYABBY3 and CsAS1, we mapped the top motifs enriched by DAP‐seq onto their respective promoters (Figure S11A,B). Y1H assays verified direct binding of CsYABBY3 to the *CsAS1* promoter (*CsAS1pro)* (Figure 6A). MST analysis further quantified this interaction, revealing a Kd of 13.4 µm (Figure 6B). Dual‐LUC assays demonstrated that CsYABBY3 activates *CsAS1pro* (Figure 6C). Consistently, qRT‐PCR analysis showed elevated *CsAS1* transcript levels in *CsYABBY3*‐OE hairy roots and reduced abundance in VIGS‐mediated *CsYABBY3*‐silenced lines (Figure 6D).

Reciprocally, both Y1H (Figure 6E) and MST (Figure 6F) confirmed that CsAS1 directly binds to the *CsYABBY3* promoter (*CsYABBY3pro*), and Dual‐LUC assays demonstrated activation of *CsYABBY3pro* by CsAS1(Figure 6G). Moreover, *CsYABBY3* expression positively correlated with *CsAS1* transcript abundance in *CsAS1‐*OE hairy roots and VIGS‐mediated *CsAS1‐*silenced plants, suggesting that *CsYABBY3pro* is a direct transcriptional target of CsAS1 (Figure 6H). Together, these results indicate that CsYABBY3 and CsAS1 mutually activate each other's transcription, forming a positive feedback regulatory loop.

### 2.7 CsYABBY3‐CsAS1 Synergistically Regulating Cannabinoid Biosynthesis

**FIGURE 7:**
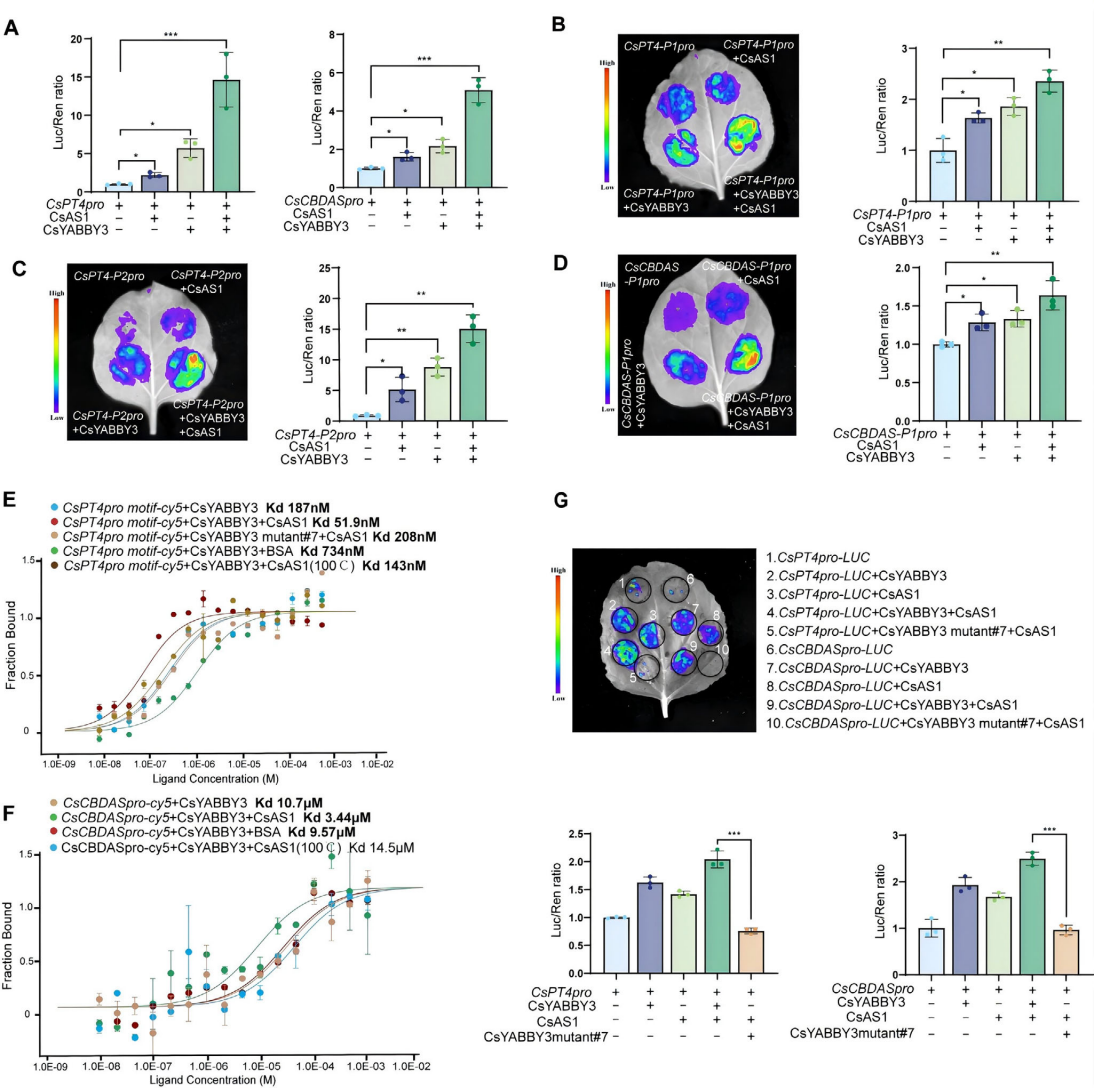
Interaction between CsAS1 and CsYABBY3 activates the transcription levels of *CsPT4pro* and *CsCBDASpro*. (A) Dual‐LUC assay showing activation of full‐length *CsPT4pro* and *CsCBDASpro* by co‐expression of CsYABBY3 and CsAS. (B‐D) Transient expression assay showing that CsYABBY3‐CsAS1 module activates *CsPT4‐P1pro* (B), *CsPT4‐P2pro* (C), and *CsCBDAS‐P1pro* (D) in *N. benthamiana* leaves. Data represent mean ± SD of three biological replicates (n = 3). (E, F) MST assays evaluating the binding of Cy5 labeled *CsPT4pro motif* (E) and *CsCBDASpro* (F) to CsYABBY3/mutant‐CsAS1 complexes. *CsPT4pro motif‐Cy5* and *CsCBDASpro‐Cy5* served as the targets, and CsYABBY3‐CsAS1 complex as the ligand. (G) Transient co‐expression of CsYABBY3‐mutant #7 and CsAS1 suppresses *CsPT4pro* and *CsCBDASpro* activities. Data are means ± SD (n = 3) from three biological replicates.

Given that CsYABBY3 alone acts as a positive regulator of cannabinoid biosynthesis, we further investigated the role of the CsYABBY3‐CsAS1 module in cannabinoid metabolism in *C. sativa*. Dual‐LUC assays revealed that co‐expression of CsYABBY and CsAS1 in *N. benthamiana* significantly activated the full‐length *CsPT4pro* and *CsCBDASpro* (Figure 7A), as well as the truncated *CsPT4‐P1pr*o (Figure 7B), *CsPT4‐P2pr*o (Figure 7C), and *CsCBDAS‐P1pro* (Figure 7D), compared to either factor alone. These results indicate that the CsYABBY3‐CsAS1 module cooperatively enhances transcription of downstream biosynthetic genes.

To assess how CsYABBY3 interface mutants affect promoter recognition in complex with CsAS1, we performed MST using complexes of mutants #1‐#8 with CsAS1 and measured binding to the *CsPT4pro* motif (Figure 7E) and *CsCBDASpro* (Figure 7F). The CsYABBY3 mutant #7‐CsAS1 complex showed a substantially reduced affinity for both promoters relative to the wild‐type CsYABBY3‐CsAS1, approaching the levels of CsYABBY3 alone or CsYABBY3 paired with inactive CsAS1. Dual‐LUC assays revealed that co‐expression of CsYABBY3 mutant #7 with CsAS1 significantly repressed *CsPT4pro* and *CsCBDASpro* activities, whereas other mutants (except #8) showed no significant differences (Figure 7G; Figure S8D). These results demonstrate that substitution at M199 residue disrupts the CsYABBY3‐CsAS1 interaction, weakening promoter binding and impairing transcriptional activation.

**FIGURE 8:**
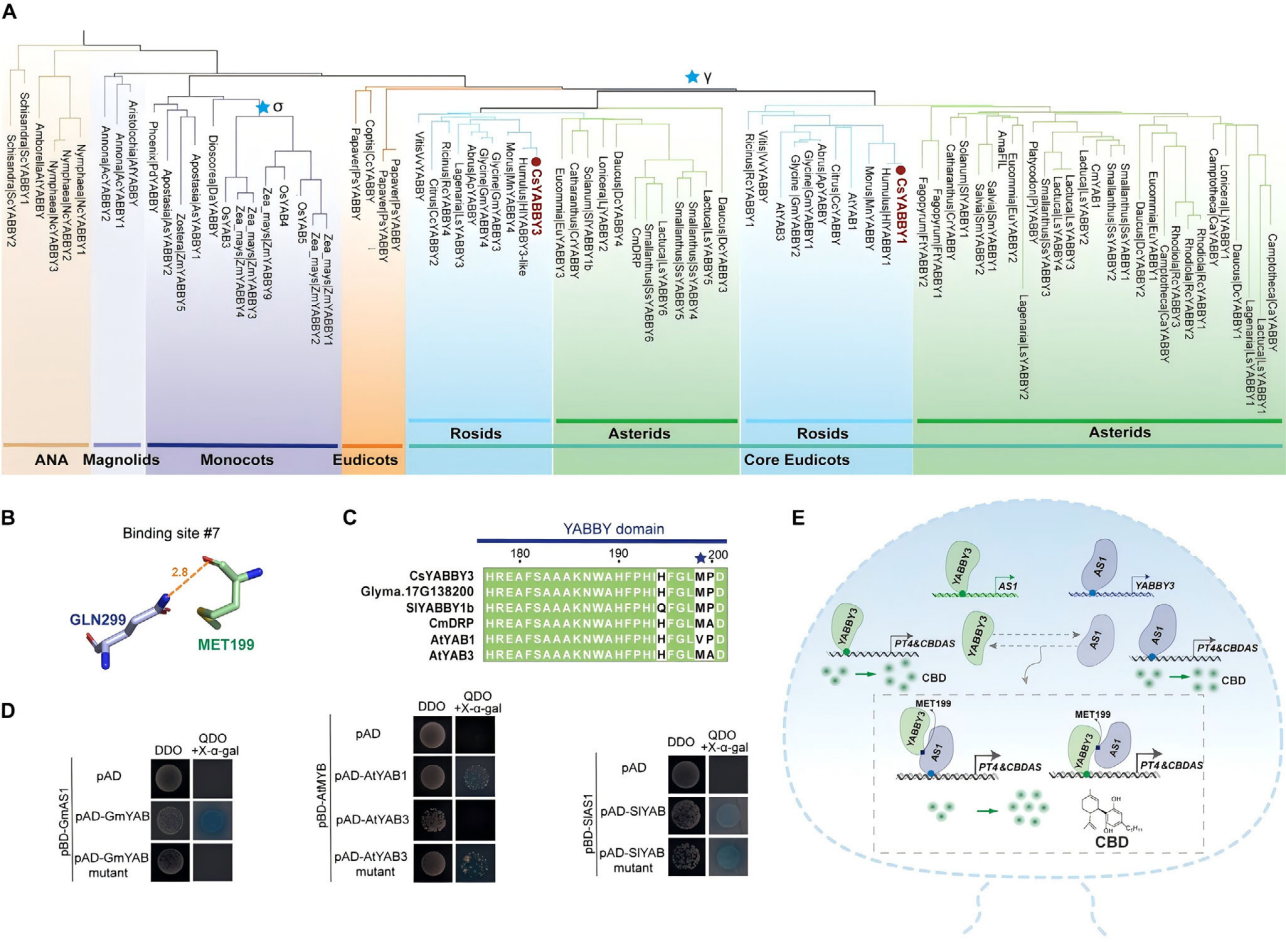
Evolutionary conservation of the YABBY‐AS1 structural interface and the proposed regulatory model for cannabinoid biosynthesis. (A) Maximum‐likelihood phylogenetic tree of the YABBYs across diverse plant lineages, highlighting the evolutionary position of *C. sativa* CsYABBY1 and CsYABBY3 (red dots). The tree was constructed with 1000 bootstrap replicates. (B) Structural modeling of active site #7 at the CsYABBY3‐CsAS1 interface, illustrating the predicted hydrogen bond involving M199 (2.8 Å). C) Sequence alignment of CsYABBY3 with homologous proteins. Cs, *Cannabis sativa*; Gm, *Glycine max*; Sl, *Solanum lycopersicum*; Cm, *Chrysanthemum morifolium*; At, *Arabidopsis thaliana*. (D) Y2H assays validating the functional conservation of the YABBY‐AS1 physical interaction across diverse core eudicots, including *G. max*, *A. thaliana*, and *S. lycopersicum*. (E) Proposed working model of the CsYABBY3‐CsAS1 regulatory module in *C. sativa*. In the nucleus, CsYABBY3 and CsAS1 form a heteromeric complex through the M199 residue. This complex synergistically binds and activates CsPT4 and CsCBDAS promoters, driving cannabinoid biosynthesis. CsYABBY3 and CsAS1 engage in reciprocal transcriptional activation: CsYABBY3 binds the CsAS1 promoter (green arrows on left) while CsAS1 binds the CsYABBY3 promoter (purple arrows on right), establishing a bidirectional positive feedback loop (dashed arrows). The M199‐mediated heterodimerization enhances DNA‐binding affinity and transcriptional potency compared to either factor alone. This self‐amplifying regulatory circuit ensures robust activation of cannabinoid biosynthetic genes, ultimately promoting CBD and related cannabinoid biosynthesis in glandular trichomes.

To explore the evolutionary significance of this interaction, we analyzed the phylogeny of the FIL/YAB3 subfamily (Figure 8A; Table S4). In core eudicots, FIL/YAB3 genes diverged into two subclades: Subclade I and Subclade II, likely as the result of their shared whole‐genome triplication‐γ. Sequence alignment revealed that the M199 residue is highly conserved across core eudicots, which structurally serves as a key docking site (Figure 8B,C). Based on the phylogenetic analysis, we cloned *AtYAB1* and *AtYAB3* from Arabidopsis (subclade I), only *GmYAB* and *SlYABBY1b* from soybean and tomato (subclade II), along with their respective AS1 homologs. Y2H assays confirmed that all these species form the FIL/YAB3‐AS1 protein complex. In soybean, as in cannabis, M199 is the critical residue for the GmYAB‐GmAS1 interaction. However, in Arabidopsis, while AtYAB1 interacts with AtAS1, AtYAB3 does not. Introduction of the double substitution (M199V/A200P) into AtYAB3 restored its interaction with AtAS1 (Figure 8D). Similarly, in tomato, the single M199 residue is insufficient to maintain the FIL/YAB‐AS1 interaction. These results suggest that the mechanism by which M199 determines FIL/YAB3‐AS1 interaction is conserved within subclade II of FIL/YAB3 in Rosids. While both subclades in core eudicots exhibit functional redundancy, they have evolved distinct mechanisms due to structural changes.

## 3 Discussion

*C. sativa*, a plant of substantial economic and therapeutic significance, synthesizes specialized cannabinoids primarily within its glandular trichomes [4]. While the biosynthetic pathways of these compounds are well understood, their regulatory networks, and in relation to trichome development remain inadequately elucidated. Here, we identify a novel transcriptional regulatory module involving FIL/YAB3 member CsYABBY3 and the R2R3‐MYB factor CsAS1 (Figure 8). These transcription factors directly activate the promoters of key biosynthetic genes (*CsPT4* and *CsCBDAS*), thereby synergistically enhancing cannabinoid production and trichome development. The increased density of both trichome types in the *CsYABBY3*‐OE tomato model suggests a general role in initiation, while the higher proportion of glandular types likely reflects a specialized preference for glandular specification through its synergy with CsAS1. This establishes a reciprocal CsYABBY3‐CsAS1 feedback loop as a core molecular switch for trichome‐specific metabolism, adding a critical regulatory layer to established biosynthetic cascades [27, 28, 29, 30].

Mechanistically, this feedback loop integrates mutual transcriptional activation with direct protein–protein interaction. DAP‐seq and functional assays confirm that CsYABBY3 and CsAS1 reciprocally bind each other's promoters (Figure S11A,B; Figure 6). This bidirectional activation ensures the robust co‐accumulation of both regulators, which subsequently physically interact to amplify the expression of shared downstream targets. Similar regulatory architectures are well‐established in plants, such as the SEP3‐AP1/AP3/AG complex in Arabidopsis [31] and the CaWRKY27b‐CaWRKY40 circuit in pepper [32]. Regarding their co‐occupancy on shared targets (*CsPT4* and *CsCBDAS*), the physical distance between the distinct CsYABBY3 and CsAS1 motifs is insufficient for a pre‐formed complex to simultaneously bridge both sites. Instead, our structural modeling and molecular docking support a mutual recruitment mechanism (Figure S11C–E). Either TF can independently anchor to its specific motif and subsequently recruit its partner to form a functional heterodimer in situ. Docking simulations indicate that complex formation does not sterically hinder DNA interaction; rather, the entire complex stably co‐binds to a single motif, enhancing overall DNA‐binding affinity without site competition.

The FIL/YAB3 subfamily phylogenetically and functionally diverges across angiosperms. In monocots, they exhibit diversified expression patterns, such as the uniform expression of rice *OsYABBY1* in specific cell layers [33, 34] and the abaxial expression of maize *ZYB9* and *ZYB14* [35]. In contrast, eudicot FIL/YAB3 members generally maintain the ancestral adaxial expression pattern, exemplified by Arabidopsis *AtYAB1* and *AtYAB*3, Antirrhinum GRAMINIFOLIA (*GRAM*), and soybean *GmFILa*, participating in lateral organ polarity and leaf morphogenesis [36, 37]. Within the FIL/YAB3 subfamily in core eudicots, subclade II represents a distinct evolutionary lineage. The FIL/YAB3‐AS1 physical interaction is conserved across various core eudicots highlights its fundamental role as an ancestral regulatory scaffold (Figure 8E). We propose that the CsAS1‐CsYABBY3 module represents an evolutionary co‐option, where this conserved developmental switch was recruited to synchronize glandular trichome differentiation with the specialized metabolic demands of cannabinoid biosynthesis. Similar recruitment of YABBY homologs for specialized metabolism has been observed in other aromatic and medicinal species, such as *M. spicata* and *A. annua* [38, 39]. Crucially, our identification of this conserved M199 residues as a requisite docking site further suggests that this biochemical interface is an inherent evolutionary feature, providing a universal molecular framework that lineages can adapt to orchestrate high‐output specialized pathways. Mutations in M199 disrupt complex formation and abolish the transcriptional activation of downstream targets, echoing the well‐known MYB‐bHLH‐WD40 (MBW) pigment model [40], where specific amino acid interactions are fundamental for functional transcriptional complexes.

The initiation and termination of this feedback loop likely involve the coordination of early hormonal fluctuations and upstream regulators, as well as the physiological maturation and senescence of the glandular trichome head [6, 28, 41]. A similar mechanism has been observed in other plants [42, 43], such as in *Arabidopsis*, where the MYC2‐DOF2.1‐MYC2 circuit has been shown to regulate leaf senescence [44]. Ultimately, these findings provide significant insights for optimizing cannabinoid production in the *C. sativa* industry. Future research should focus on expanding the network of regulators that interact with this complex to provide a more comprehensive understanding of the transcriptional landscape in *C. sativa*. Moreover, while our study establishes a robust transcriptional framework, future endeavors aimed at characterizing the endogenous protein stability and post‐translational dynamics of the CsYABBY3‐CsAS1 complex will further refine the spatiotemporal precision of this regulatory axis. It will also be important to explore how environmental factors, such as light, temperature, and nutrient availability, influence the expression of CsYABBY3 and CsAS1, as these factors are known to affect trichome density and cannabinoid content in plants. Additionally, validating the role of the CsYABBY3‐CsAS1 feedback loop under field conditions will be crucial for translating these findings into practical agricultural applications.

## 4 Experimental Section

### 4.1 Plant Materials and Growth Conditions

Previously described cannabis organ transcriptomes from multiple cultivars established by our group were used [45]. *C. sativa*, *A. thaliana*, and *N. benthamiana* plants were sourced from the greenhouse of Northeast Forestry University. Plants were grown under a 16‐h light/8‐h dark photoperiod at 23°C.

### 4.2 RNA‐seq and Transcriptome Analysis

Total RNA was extracted from flowers, bracts, sugar leaves, leaves, stem, roots, seeds of *C. sativa* cultivar“Diku” using the Quick RNA extraction kit (Huayueyang, Beijing, China). Samples with RNA integrity number of > 8 were retained for sequencing. Illumina sequencing was performed at BGI Genomics Co. Ltd. (Beijing, China). and RNA‐seq data were processed, assembled, and annotated. All the assembled transcripts were aligned to six databases: NR, Swiss‐Prot, Pfam, COG, GO, and KEGG to obtain annotation information. Gene expression was quantified using Fragments Per Kilobase Million values.

### 4.3 Standards and Chemical Reagents

OA (CAS: 508‐02‐1), CBD (CAS: 13956‐29‐1), CBDA (CAS: 1244‐58‐2), CBG (CAS: 25654‐31‐3); CBGA (CAS: 25555‐57‐1), and GPP (CAS: 104715‐14‐2) standards were obtained from Sigma–Aldrich (USA).

### 4.4 MALDI‐Imaging

Bracts, sugar leaves, and leaves were mounted on indium tin oxide (ITO)‐coated glass slides. To enhance ionization, samples were treated with a matrix solution containing 10 mg/mL DHB in a 1:1 (v/v) mixture of methanol and water. MALDI‐imaging was performed with a spatial resolution of 15 µm using MirionV3 software. Other experimental parameters were based on previous protocols published by our group [46].

### 4.5 Phylogenetic Analysis and Sequence Alignment

Amino acid sequences of CsYABBY3 protein and its homologs were retrieved from the NCBI database using BLAST (https://blast.ncbi.nlm.nih.gov/). Homology matching was performed using BLASTP searches and the ‘Blast Several Sequences to a Big Database' function in TBtools, with parameters set to an E‐value < 10^−^^5^ and redundant sequences removed, ultimately identifying all YABBY gene family members in other species. Multiple amino acid sequences were aligned using ClustalW, and phylogenetic trees were constructed using Maximum likelihood method implemented in the MEGAX program with 1000 bootstrap replicates [47, 48].

### 4.6 Total RNA Isolation and qRT‐PCR

RNA was extracted from flash‐frozen samples using the FastPure Plant Total RNA Isolation Kit (Vazyme, China). cDNA synthesis and qRT‐PCR was performed using ReverTra Ace qPCR RT Master Mix with gDNA Remover (TOYOBO, Japan) and ChamQ Universal SYBR qPCR Master Mix (Vazyme, China) [49, 50]. Relative gene expression was calculated using the 2^−△△CT^ methods as previously described [51]. Primers used for qRT‐PCR are listed in Table S3.

### 4.7 Gene and Promoter Cloning

Open reading frames (ORFs) of *CsAS1, CsYABBY3*, and homologs from other species were amplified using KOD One PCR Master Mix (TOYOBO, Japan) from corresponding cDNA templates using gene‐specific primers (Table S3). Promoter regions of CBDA biosynthetic genes and TFs were amplified from genomic DNA of the cannabis cultivar Diku.

### 4.8 Subcellular Localization

Coding sequences of CsYABBY3 and CsAS1 were fused in‐frame to eGFP in the pEAQ‐HT‐eGFP vector. Constructs were transformed into *Agrobacterium tumefaciens* strain GV3101 (Psoup) and infiltrated into *N. benthamiana* leaves. GFP fluorescence was imaged on a Zeiss LSM510 META confocal laster‐scanning microscope after 24 h in darkness followed by 48 h under light.

### 4.9 Prediction of YABBY Transcription Factor Binding Sites

Promoter sequences of *CsPT4* and *CsCBDAS* were scanned for YABBY TF binding sites using the JASPAR 2024 database (http://jaspar.genereg.net) [52]. Sites with relative score above 0.90 were mapped to genomic positions.

### 4.10 RNA in Situ Hybridization

*C. sativa* vegetative leaves were fixed in ice‐cold 1 × PBS with 4% paraformaldehyde/4% DMSO, vacuum‐infiltrated for 3 min, and post‐fixed with replaced fixative overnight at 4°C. Samples were dehydrated, cleared, and embedded in Paraplast (Sigma–Aldrich, cat. no. P3683); 8 µm sections were cut using a Leica RM2235 microtome. Gene‐specific 400–1000 bp fragments (Table S3) were cloned bidirectionally into the *pEASY*‐Blunt Zero cloning vector (TransGen Biotech, CB501) to generate antisense/sense probes. The digoxigenin‐labelled (Roche, 11277073910) antisense and sense probes were transcribed with T7 RNA polymerase (Promega, P4074). Sections were dewaxed, treated with proteinase K, dehydrated, and incubated in probe buffer for 12 h at 50°C in a humidified box. The sections were then incubated with anti‐digoxin antibody (Roche, 11093274910) for 2 h at 25°C, and developed with NBT/BCIP reaction solution (Roche, 11681451001) for 36–48 h at 25°C in a dark humidified box. The images were acquired by bright‐field microscope (Olympus BX‐50).

### 4.11 SEM

The middle part of the first leaf from the third internode was surveyed using a HITACHI UHR FE‐SEM SU8010 Series scanning electron microscope (Hitachi, Tokyo, Japan). Trichome counts were determined using the IMAGEJ software (https://imagej.nih.gov/ij/download.html).

### 4.12 Cannabinoid Extraction and LC‐MS/MS

Lyophilized samples (100 mg) were extracted with 1 mL 95% methanol, sonicated at room temperature for 30 min, and incubated at 4°C overnight. Supernatants after 10 000 g for 15 min centrifugation (twice extractions) were pooled and filtered through 0.22 µm for LC‐MS/MS analysis on an Agilent UPLC 1290II‐G6400 triple quadrupole mass spectrometer. Separation used an MS/MS spectra were obtained in negative ionization mode using a C18 column (Eclipse Plus C18 column (2.1 mm × 100 mm, 1.8 µm) in negative ionization mode. The mobile phases were 0.1% (v/v) formic acid in water (A) and methanol (B) under a linear gradient program: 0/70, 2/70, 10/100, 13/100, 14/95, and 15/95 (min/%B). Cannabinoid content was calculated using th formulas: total CBD = CBD + 0.877 × CBDA, total CBG = CBG + 0.878 × CBGA [53, 54, 55].

### 4.13 Dual‐Luciferase Reporter Assay

The coding regions of *CsYABBY3* and *CsAS1* were cloned into pGreenII62‐SK to generate the effectors. The promoter sequence of *CsPT4* and *CsCBDAS* was cloned into the pGreenII 0800‐LUC for reporter constructs. Agrobacterium‐mediated transient expression was performed in *N. benthamiana* leaves, and LUC activity was visualized using the NightSHADE LB 985 Plant Imaging System (Berthold Technologies, Germany) and subsequently measured using the Dual‐Luciferase Reporter Assay System E1910 (Promega, USA). The LUC/REN ratio was normalized relative to the corresponding controls the experiments were carried out at least three times.

### 4.14 DAP‐seq (DNA Affinity Purification Sequencing) Assay

DAP‐seq was conducted following previously published protocols [56]. Briefly, *C. sativa* genomic DNA was isolated and added to an affinity‐purified CsYABBY3 and CsAS1 protein linked to a Halo tag. The binding portion was eluted, amplified, sequenced, and mapped to the reference genome. Genes corresponding to the detected peaks were identified and subjected to GO and KEGG analysis.

### 4.15 Protein Expression and Purification

*CsYABBY3* or its mutant (CsYABBY3‐mutant #1∼8) and *CsAS1* were cloned into the pCOLD‐TF expression vector, and recombinant plasmids were transferred into the *E. coli* BL21 (DE3). After induction, proteins were purified using a nickel column and eluted with 200 mm imidazole. Protein concentrations were determined using Bradford reagents (Transgen, Beijing, CN), and SDS–PAGE was performed for confirmation.

### 4.16 MST Assay

The promoter of *CsPT4* and *CsCBDAS* were conjugated with the Cy5 fluorophore. *CsYABBY3* and its mutants were fluorescently labeled using the Monolith Protein Labeling Kit RED‐NHS second Generation (Nanotemper Technologies, Germany) according to the manufacturer's protocol. For DNA‐protein interaction, the Cy5 labeled DNA fragments served as targets, and proteins served as ligands; for protein‐protein interaction, the NHS labeled CsAS1 was used as the targets, and CsYABBY3 and its mutants served as the ligands. Binding assays were performed in PBS buffer, and data were analyzed using MO. Affinity Analysis v2.2.4.

### 4.17 Isothermal Titration Calorimetry (ITC) Assay

ITC was performed using an iTC200 microcalorimeter (MicroCal). Interaction was performed in a buffer (20 mm Tris–HCl pH7.5) at 25°C. CsYABBY3 (200 µm) was titrated into the cell containing 200 µl of CsAS1 (20 µm) or EV (20 µm), respectively. Binding curves were generated by plotting the heat change of the binding reaction, and the data were analyzed using MicroCal Analysis Software.

### 4.18 LCI Assay

Full‐length CDSs of CsAS1 and CsYABBY were fused to N‐terminal and C‐terminal luciferase constructs, respectively. These constructs were co‐expressed in *N. benthamiana* leaves via *Agrobacterium* infiltration. For LCI, 1 mm of potassium salt of D‐fluorescein (Promega, USA) in PBS was sprayed onto the leaf surface and incubated for 7 min in the dark. LCI imaging was performed using the NightSHADE LB 985 Plant Imaging System (Berthold Technologies, Germany).

### 4.19 Y1H Assay

Y1H assays were performed using the Machmarker Gold Yeast One‐Hybrid System (Clontech, Japan). Promoters of *CsPT4* and *CsCBDAS* were cloned into pAbAi and the constructs were transformed into the Y1H Gold yeast to generate the bait strains. Full‐length CDSs of CsYABBY3 and CsAS1 were cloned into pGADT7 vector and transformed into the bait yeast strains. Yeast transformations were selected on SD‐Ura/‐Leu agar medium plates containing 100–900 ng/m lAureobasidin A.

### 4.20 Yeast Screening Library and Y2H Assay

Full‐length CDS of *CsYABBY3* was cloned into the pGBKT7 vector, which served as the bait to screen potential interacting proteins from pGADT7‐ *C. sativa* cDNA library constructs. The positive transformants were sequenced and mapped to the reference genome. Y2H assays were performed based on the Matchmaker Gold Yeast Two‐Hybrid System (Clontech) to confirm protein interaction point‐to‐point. The pGADT7‐CsAS1 and pGBKT7‐CsYABBY3 constructs were co‐transformed into Y2H Gold yeast strain. Yeast transformants were selected on QDO (SD/‐Trp/‐Leu/‐His/‐Ade) with X‐α‐Gal.

### 4.21 Co‐IP Assay

*Pro35S:CsYABBY3‐GFP* and *Pro35S:CsAS1‐HA* constructs were introduced into *A. tumefaciens* strain AGL1 and co‐infiltrated into *N. benthamiana* leaves. Total protein was extracted in ice‐cold buffer containing 50 mm pH 8.0 Tris‐MES, 150 mm NaCl, 1 mm MgCl_2_, 500 mm sucrose, 10 mm EDTA, 5 mm DTT, 1 mm PMSF, and 1 mm Cocktail. Immunoprecipitation was performed with anti‐GFP Magnetic Beads (share‐bio, Shanghai) to pull down CsYABBY3‐GFP complexes; co‐precipitated CsAS1‐HA was detected by immunoblotting with anti‐HA antibody. Input and immunoprecipitated fractions were probed with anti‐GFP and anti‐HA.

### 4.22 Transient Overexpression and RNAi in Cannabis Leaves

For transient overexpression, full‐length CDS of *CsYABBY3* and *CsAS1* were cloned into a *35S*‐driven overexpression vector carrying the RUBY reporter to generate *CsYABBY3*‐OE and *CsAs1*‐OE constructs. For RNAi, ∼300 bp gene‐specific fragments of *CsYABBY3* or *CsAS1* were inserted into the pHANNIBAL vector. All constructs were introduced into the *Agrobacterium tumefaciens* strain AGL1 and vacuum‐infiltrated into *C. sativa* leaves. Infiltrated plants were maintained for 5–7 days prior to sampling for transcript and metabolite analyses.

### 4.23 Stable Transformation via Hairy Roots

Hairy root transformation followed published procedures [57]. *Agrobacterium rhizogenes* strain K599 harboring *CsYABBY3*‐OE or *CsAs1*‐OE constructs was used to inoculate Cannabis seedlings after removal of approximately two‐thirds of the root system. After ∼4 weeks of cultivation, RUBY‐positive (red) transformed hairy roots were harvested for gene expression and cannabinoid quantification.

### 4.24 VIGS

Gene‐specific **∼**300‐bp fragments of *CsYABBY3* and *CsAS1* were cloned into pTRV2 to generate pTRV2‐*CsYABBY3* and pTRV2*‐CsAS1*. *A. tumefaciens* AGL1 carrying pTRV1 and respective pTRV2 constructs (or pTRV2‐*PDS* as a visual indicator) was infiltrated into young cannabis seedlings leaves. At this developmental stage, the leaves primarily bear non‐glandular trichomes and sessile glandular trichomes. Therefore, the analysis was primarily focused on the transcript abundance of CsYABBY3 and the resulting metabolic profile. Plants were maintained for 3–4 weeks before qRT‐PCR and LC‐MS analyses.

### 4.25 AsODN Assay

AsODNs targeting *CsYABBY3* and *CsAS1*, along with corresponding sense ODN controls, were designed using SFold (https://sfold.wadsworth.org/cgibin/soligo.pl). Healthy *Cannabis* leaves at comparable developmental stages were immersed in 10 µm ODNs diluted in 200 mm sucrose and incubated in the dark at 23°C with 60%–70% humidity for 48 h. Following treatment, the leaves were collected for subsequent analyses.

### 4.26 Tomato Transformation

The full‐length CDS of *CsYABBY3* was inserted into 35S‐driven pCAMBIA1300 vector and introduced into *A. tumefaciens* strain GV3101. Stable transformation of tomato (*S. lycopersicum* cv. MicroTom) was performed following the published method [58]. Transgenic lines were selected on hygromycin‐containing medium and verified by PCR. For further functional analysis, T1 transgenic plants at the fruiting stage were selected to observe leaf epidermal trichomes, and leaf and fruit tissues were collected to determine the composition and content of terpenoids.

### 4.27 Determination of Total Terpenoids

Approximately 0.1 g of freeze‐dried and ground transgenic tomato leaf or fruit tissue was accurately weighed and extracted with 10 mL of methanol by ultrasonication for 30 min. The extract was kept at 4°C overnight and centrifuged to obtain the supernatant, which was filtered for analysis. A standard curve was generated using oleanolic acid as the reference compound ranging from 25 to 400 µg. Each tube was treated with 200 µL of 5% vanillin‐acetic acid and 400 µL of concentrated sulfuric acid, followed by incubation in a 60°C water bath for 15 min. Five milliliters of glacial acetic acid was added, and absorbance was measured at 543 nm. The same procedure was applied to 500 µL of the sample extracts, and total terpenoid content was calculated from the standard curve. Samples without oleanolic acid were used as blank controls.

### 4.28 Molecular Docking

Protein structure models of CsYABBY3 and CsAS1 were predicted using Alphafold3 software (https://alphafoldserver.com/). Molecular docking analyses were subsequently performed, and the results were visualized and analyzed by using PyMOL [59].

### 4.29 Statistical Analysis

All experiments were independently repeated at least three times, and each experiment possessed at least three biological replicates, unless indicated otherwise. Statistical analyses were performed using GraphPad Prism 8.0. Comparisons between two groups were conducted using unpaired two‐tailed student's *t*‐test. Statistical significance was indicated as follows: ****p < 0.0001, ***p < 0.001, **p < 0.01, *p < 0.05; ns, no significance (*P* >0.05).

### 4.30 Accession Numbers

Sequence data from this article can be found in the National Center for Biotechnology Information repository (https://www.ncbi.nlm.nih.gov/bioproject/) under the following accession numbers: *CsYABBY1* (Cs_C05H1G183830), *CsYABBY2* (Cs_C07H1G284650), *CsYABBY3* (Cs_C01H1G013660), *CsYABBY4* (Cs_C09H1G358710), *CsYABBY5* (Cs_C02H1G050890), *CsAS1* (Cs_C09H1G365360), *CsPT4* (Cs_C10H1G408210), *CsCBDAS* (Cs_C06H2G258260), *CsAAE* (Cs_C04H1G135240), *CsGPPS* (Cs_C02H1G047885), *CsOLS* (Cs_C09H1G367230), *CsOAC* (Cs_C08H2G335610). RNA‐seq data of this study have been deposited in the PRJCA029174.

## Author Contributions

Wei Sun, Zhichao Xu, Ming Luo, and Shilin Chen conceived the project and designed the research plan. Xuewen Zhu, Yaolei Mi, Pucheng Fan, and Yiming Zhang performed most of the experiments. Xue Cao, Weiqiang Chen, Wei Yang, Huihua Wan, and Xiangxiao Meng analyzed the data. Jing Wang, Jun Li, Shuo Shen, Mingkun Huang and Xiaoyu Zhang contributed materials and/or analysis tools. Xuewen Zhu, Yaolei Mi, and Wei Sun wrote the paper. All authors read and approved the final manuscript.

## Conflicts of Interest

The authors declare no conflicts of interest.

## Supporting information




**Supporting File**: advs75055‐sup‐0001‐SuppMat.pdf.

**Supporting Table 1**: advs75055‐sup‐0002‐Supplementary Table 1.pdf.

**Supporting Table 2**: advs75055‐sup‐0003‐Supplementary Table 2.pdf.

**Supporting Table 3**: advs75055‐sup‐0004‐Supplementary Table 3.pdf.

**Supporting Table 4**: advs75055‐sup‐0005‐Supplementary Table 4.pdf.

## Data Availability

The data that supports the findings of this study are available in the supplementary material of this article.
