# Supplementary material for: A Novel CsYABBY3‐CsAS1 Feedback Loop Coordinates Trichome Differentiation and Cannabinoid Biosynthesis in Cannabis sativa L
Source: Adv Sci (Weinh). 2026 Apr 2;13(34):e75055. doi: 10.1002/advs.75055 (PMC13285160; doi:10.1002/advs.75055)
Supplement: Supplementary file 1 — Supporting File: advs75055‐sup‐0001‐SuppMat.pdf. [file ADVS-13-e75055-s005.pdf]

# Supplemental Information

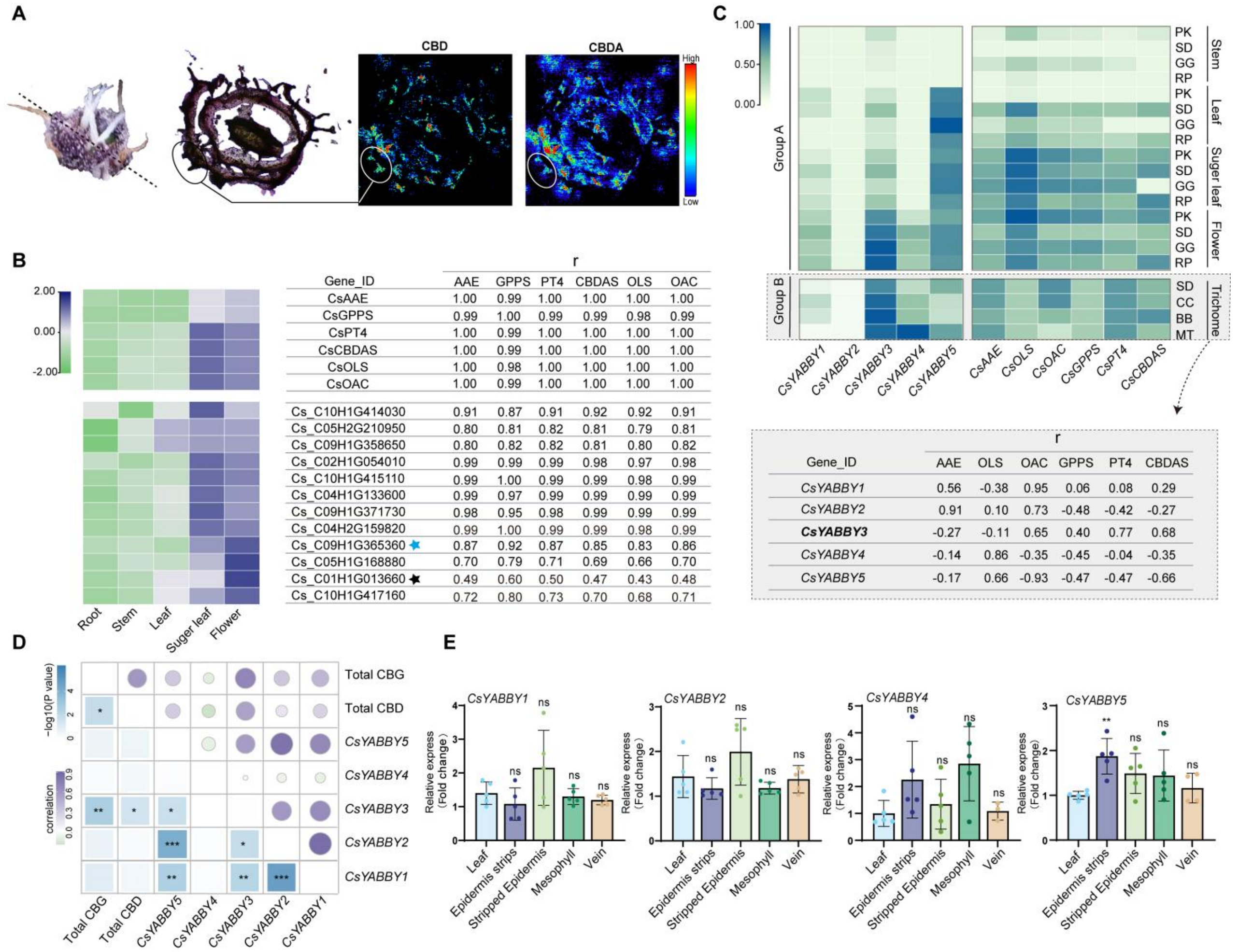


**Supplemental Figures S1. Identification and expression pattern of *CsYABBY3* in *C. sativa*.** A) Transverse section of the bract and MALDI MSI images showing the relative distribution of major cannabinoids in bract of *C. sativa*, including CBD (m/z: 313.2173), CBDA (m/z: 357.2070). All MALDI MSI images were obtained by extracting the ionized species at the M +e-H adduct. Glandular trichomes are marked with circles in the bright-field and mass spectrometry imaging images. B) Pearson correlation coefficient between the expression levels of *C. sativa* annotated TFs and key enzymatic genes (*CsAAE*, *CsGPPS*, *CsPT4*, *CsCBDAS*, *CsOLS*, *CsOAC*) in the cannabinoid biosynthesis pathway. Heatmap of TFs gene expressions depicted by z-scores calculated from $\log_{10}$ (FPKM + 1). *CsYABBY3* (Cs_C01H1G013660) and *CsAS1* (Cs_C09H1G365360) are highlighted in black and blue star, respectively. C) Expression analysis of *CsYABBYs* and cannabinoid biosynthesis key enzymes across diverse genetic backgrounds. Group A (cultivars: PK, SDA, GG and RP) demonstrated flowers/ sugar leaves specificity in our laboratory samples. Group B (SD, CC, BB, and MT) utilizes a public dataset to confirm glandular trichome specificity. The consistent high expression across these two independent groups underscores the evolutionary conservation of *CsYABBY3* in *C. sativa*. D) Correlation analysis of the transcription abundance of *CsYABBYs* with contents of total CBD and total CBG in the cannabinoids biosynthetic pathway in *C. sativa*. E) qPCR analysis of the expression level of *CsYABBYs* in various leaf tissues. At least three independent replicates were performed. *****P* < 0.0001, ****P* < 0.001, ***P* < 0.01, **P* < 0.05, and ns, no significance based on one-way ANOVA.

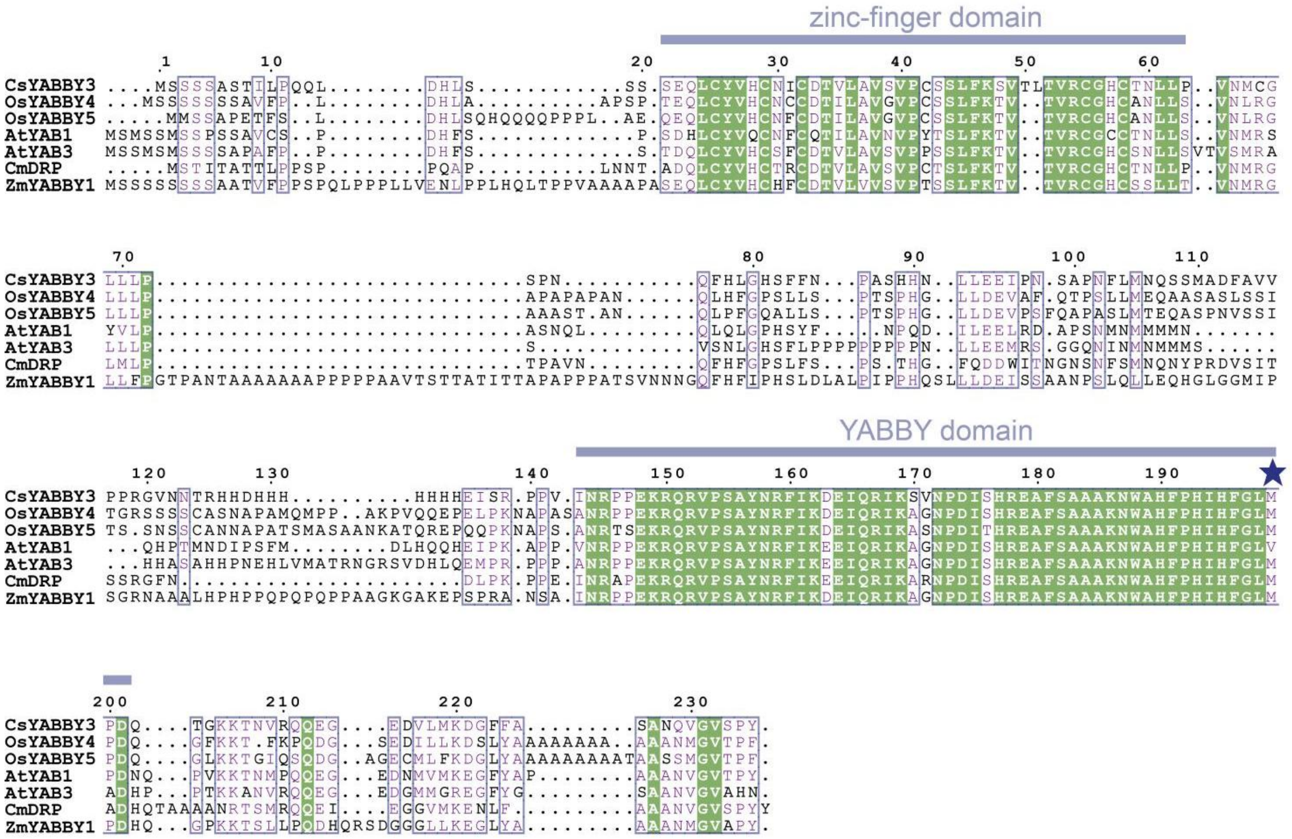


**Figure S2.** Protein sequence alignment of YABs from *Arabidopsis thaliana*, *Glycine max*, *Zea mays*, and *Oryza sativa*.The zinc finger domain and YAB domain were indicated by the purple lines.

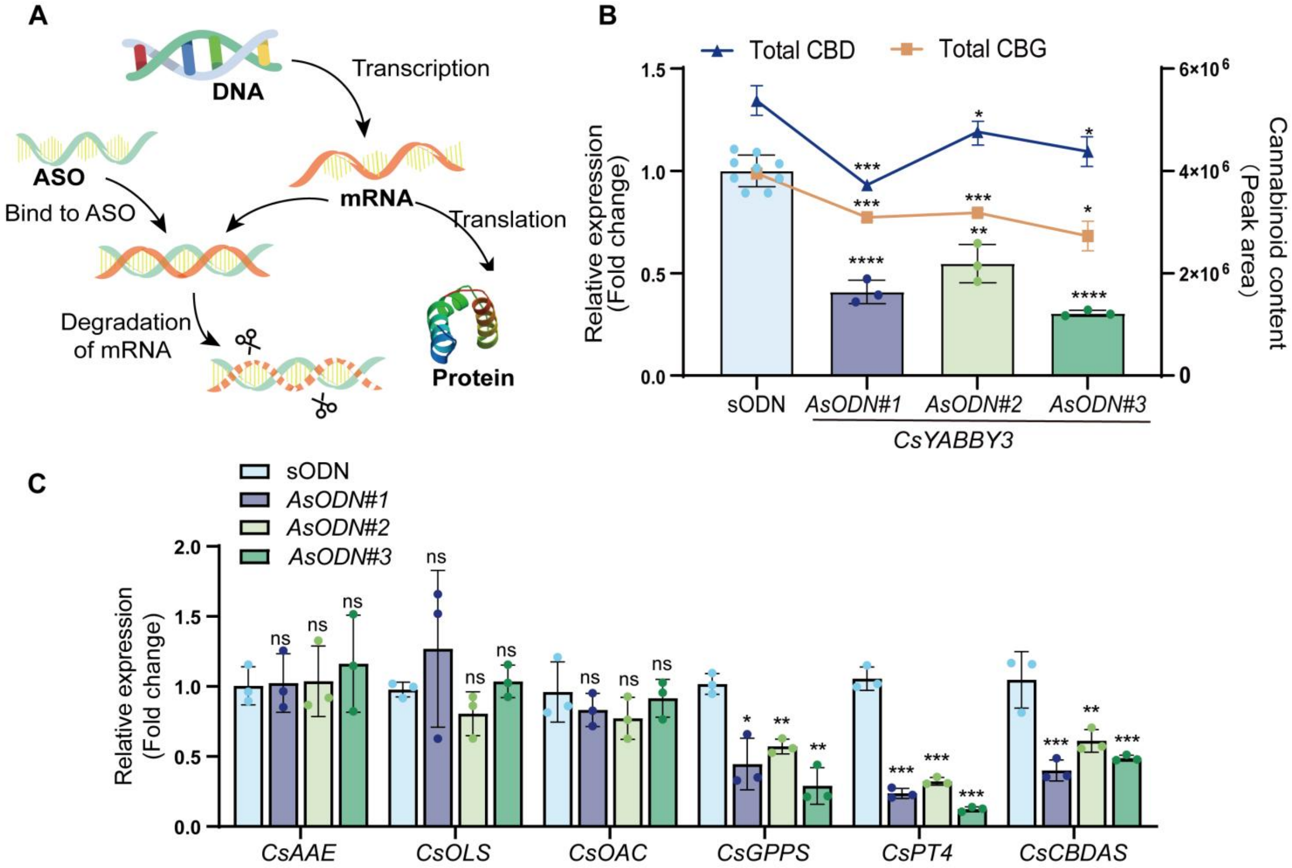


**Figure S3. Establishment of *CsYABBY3*-suppressed via Antisense Oligodeoxynucleotide (AsODN) in *C. sativa.*** A) Schematic diagram illustrating the principle of the AsODN-mediated gene knockdown approach for *CsYABBY3* in *C. sativa.* B) The total CBD/CBG content and relative expression of CsYABBY3 in AsODN-treated lines. C) qRT-PCR analysis of genes involved in cannabinoid biosynthesis in *CsYABBY3*-suppressed lines. Data in (B and C) represent means ± SD of three independent experiments. Statistical significance was determined by Student's *t*-test compared to the control: ***$P < 0.001$, **$P < 0.01$, *$P < 0.05$.

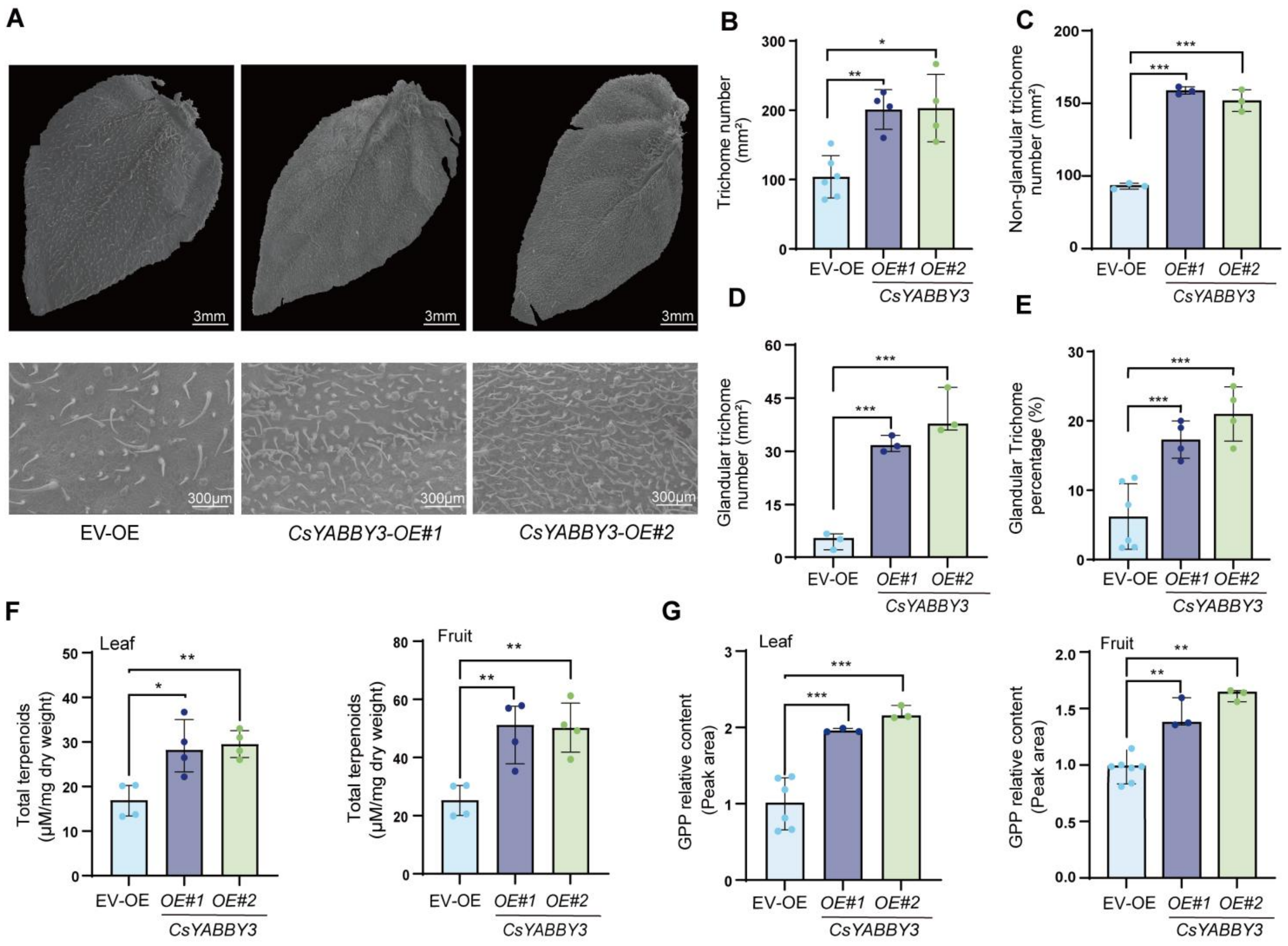


**Figure S4. Heterologous overexpression of *CsYABBY3* in tomato.** (A) Representative SEM images of leaves from 35S: *CsYABBY3* transgenic tomato plants. Scale bars, 3 mm and 300 μm. (B-E) The trichome number (B), non-glandular trichome number (C), glandular trichome number (D), and glandular trichome percentage (E) in the leaves of *CsYABBY3*-OE tomato plants. (F-G) Total terpenoid content (F) and geranyl diphosphate (G) in CsYABBY3-OE tomato leaves and fruits. Data represent mean ± SD (n = 3 biological replicates). Statistical significance was determined by Student's *t*-test compared to the control: $P < 0.05$, *$P < 0.01$, **$P < 0.001$; ns, not significant.

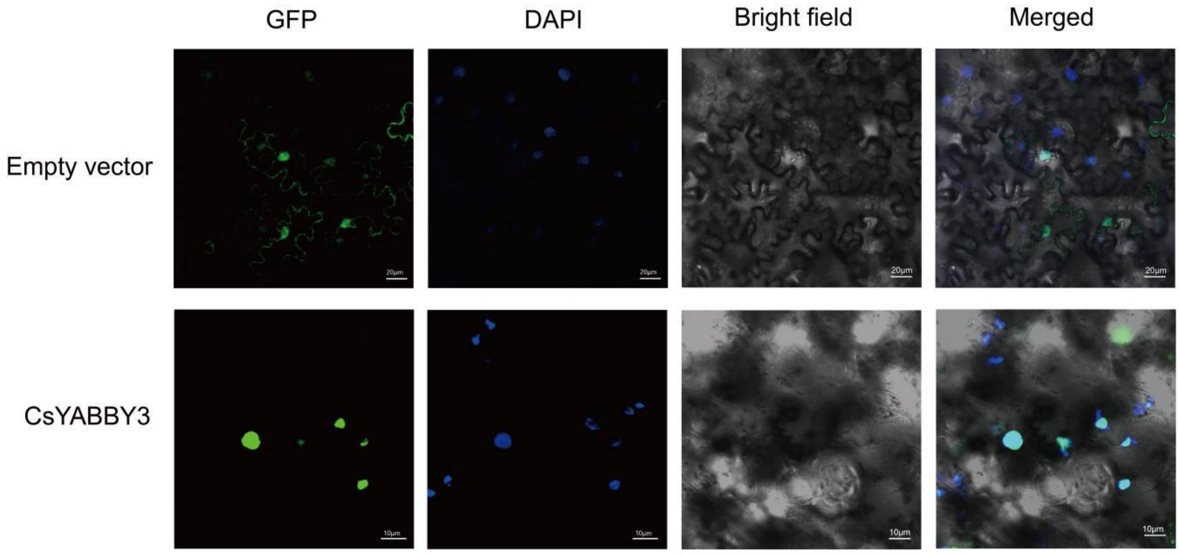


**Figure S5.** Subcellular localization of CsYABBY3 in *Nicotiana benthamiana* leaf cells. Bar, 10μm.

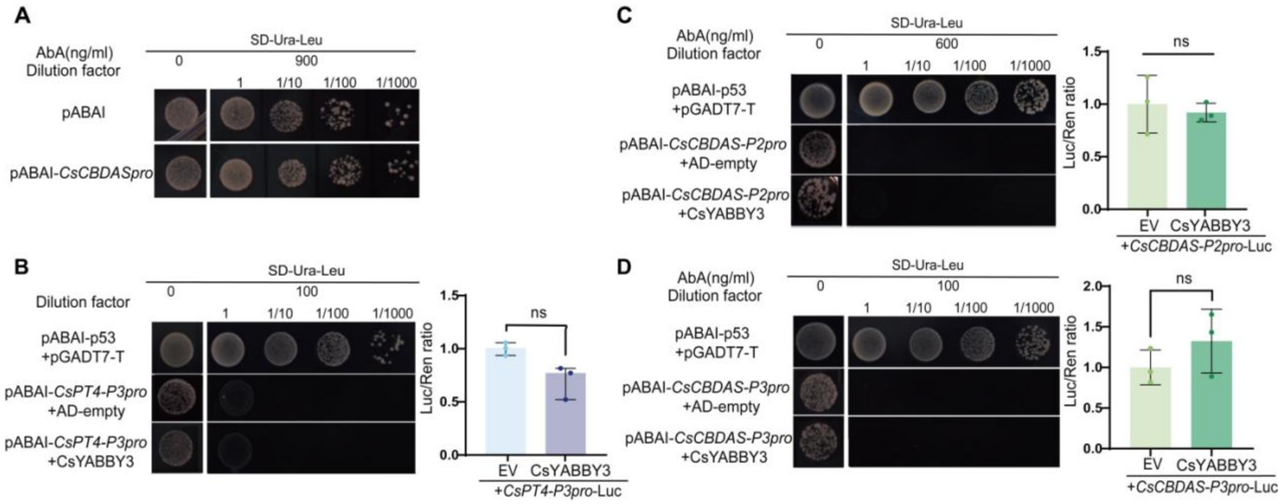


**Figure S6. Self-activation assays and transient expression analysis of *CsPT4* and *CsCBDAS* promoters.** A) Self-activation analysis of full-length *CsCBDAS* promoters in yeast. B) Self-activation analysis of truncated *CsPT4* promoter fragments (*CsPT4-P1~P3*). C) Self-activation analysis of truncated *CsCBDAS* promoter fragments (*CsCBDAS-P1~P3*). B-D) Y1H and transient dual-luciferase assay testing CsYABBY3 does not activate *CsPT4-P3pro* or *CsCBDAS-P2/P3pro*. At least three independent replicates were performed, ns, no significance based on Student's *t*-test.

A

| sample | total reads | reads mapped | reads mapped and paired | reads unmapped | map ratio |
|---|---|---|---|---|---|
| YABBY3_1 | 50313402 | 49219084 | 49146308 | 1094318 | 0.97825 |
| YABBY3_2 | 52948130 | 52024059 | 51959916 | 924071 | 0.982548 |
| YABBY3_Input | 43882938 | 43593534 | 43522534 | 289404 | 0.993405 |

B

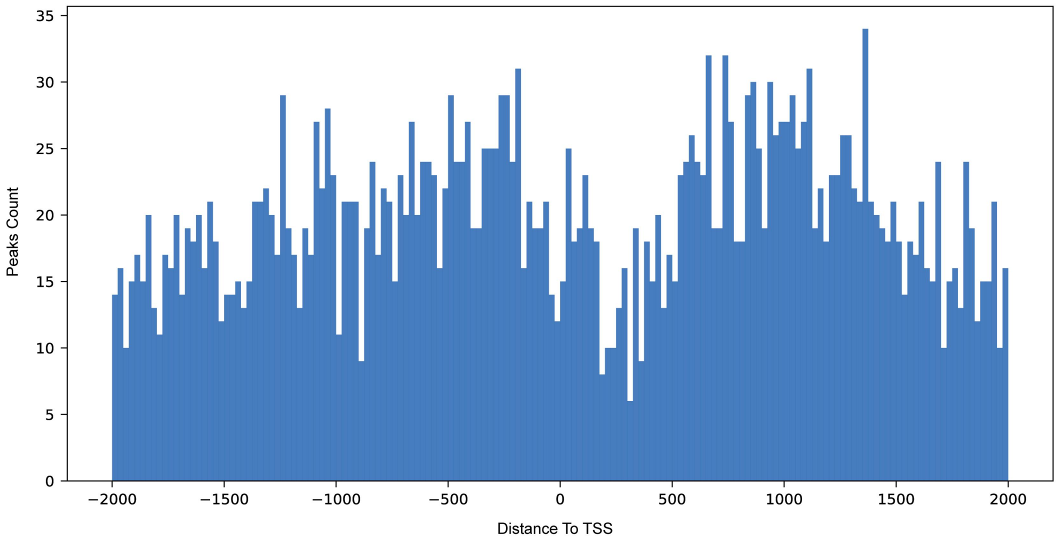


C

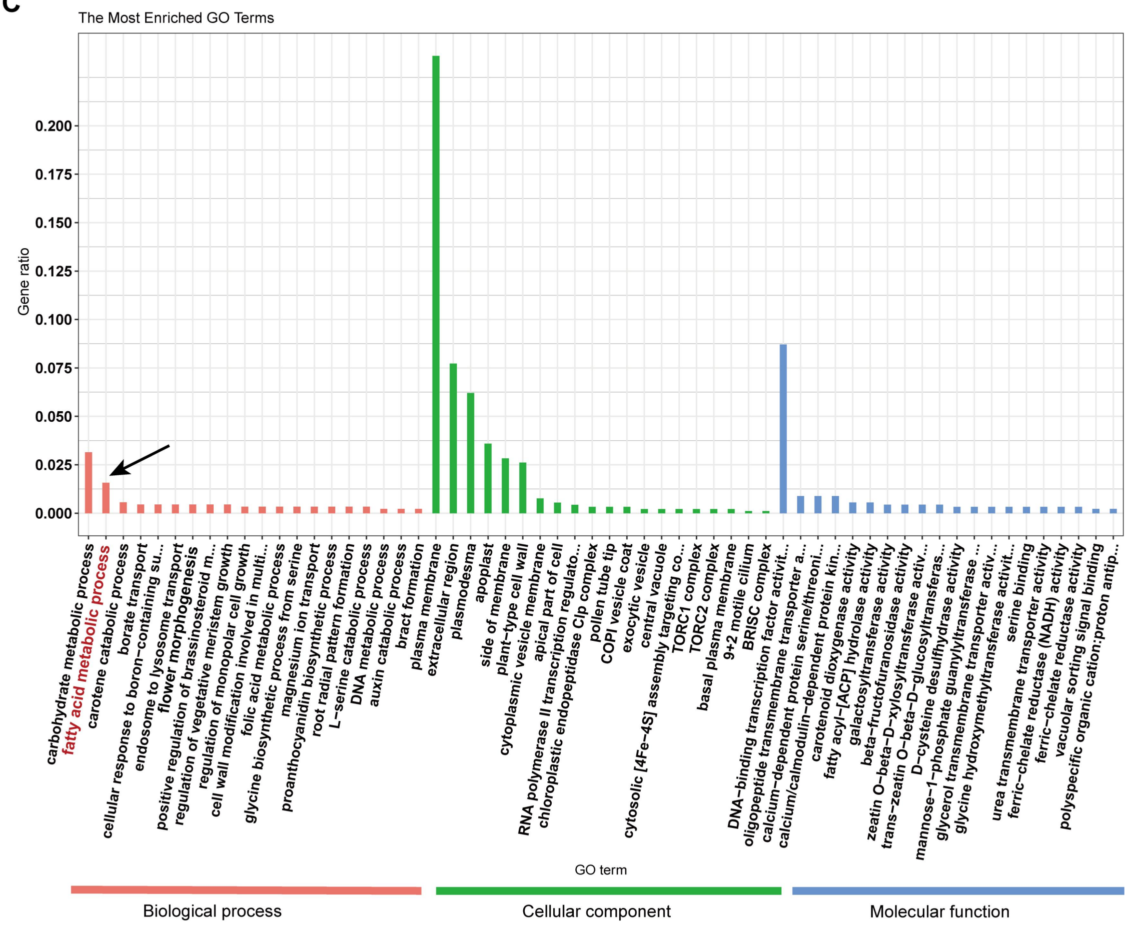


**Figure S7. DAP-seq analysis identifying potential target genes of CsYABBY3.** A) Statistics of sequence mapping for DAP-seq libraries. B) Genomic distribution of CsYABBY3 binding peaks relative to the transcription start site (TSS). C) GO enrichment of peak-associated genes across biological process, cellular component, and molecular function. The fatty acidmetabolic process was highlighted.

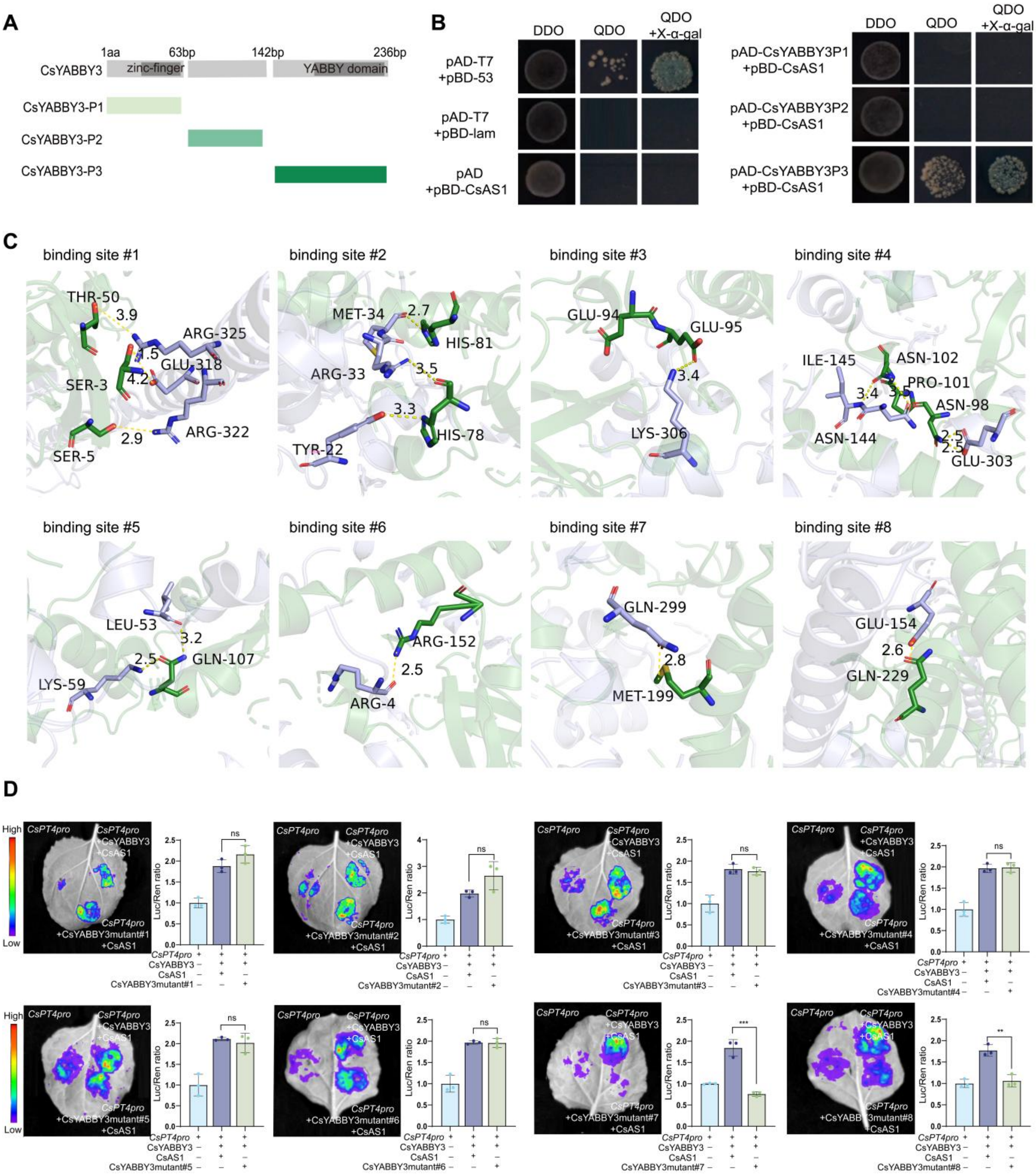


**Figure S8. Interaction mechanism between CsYABBY3 and CsAS1.** A) Schematic of CsYABBY3 protein truncations: CsYABBY3-P1 (1-63aa), CsYABBY3-P2 (64-142aa), and CsYABBY3-P3 (143-239aa). B) Y2H assays showing protein-protein interactions between CsYABBY3-P1-P3 and CsAS1. C) Enlarged view of the CsAS1 interface with CsYABBY3-mutan t#1-#8, indicating predicted hydrogen bonds (#1-#8) and bond lengths (Å). D) Transient co-expression of CsYABBY3-mutant #1-#8 with CsAS1 showing effects on the transcriptional activity of the C*sPT4pro*. Representative images at 72 h post-infiltration are shown. ****P* < 0.001, ***P* < 0.01, ns, no significance, *P* > 0.05, Student's *t*-test). Data are means ± SD; n=3 biological replicates.

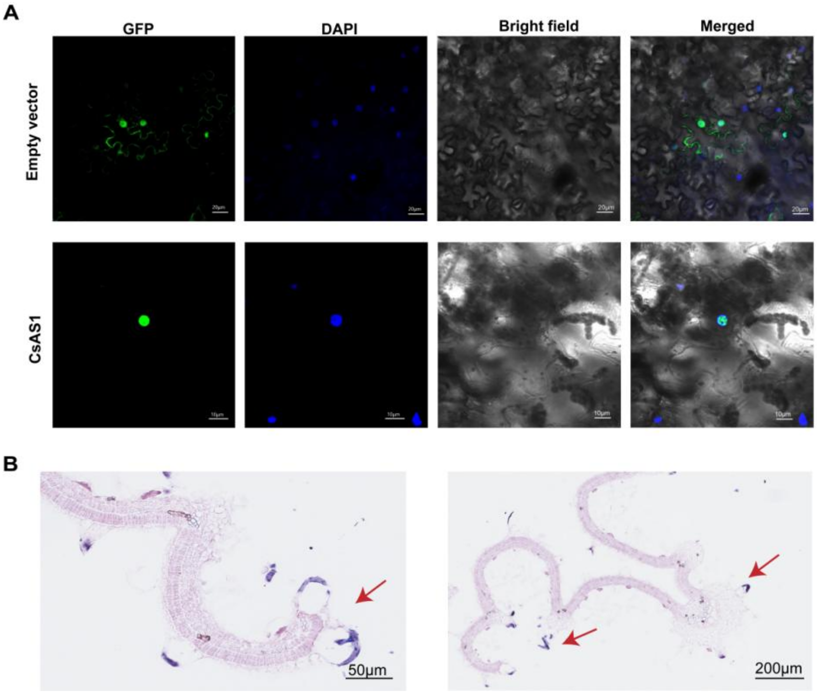


**Figure S9. *CsAS1* expression pattern.** A) Subcellular localization of CsAS1. B) RNA in situ hybridization of *CsAS1*. Arrowheads indicate sites of transcript accumulation.

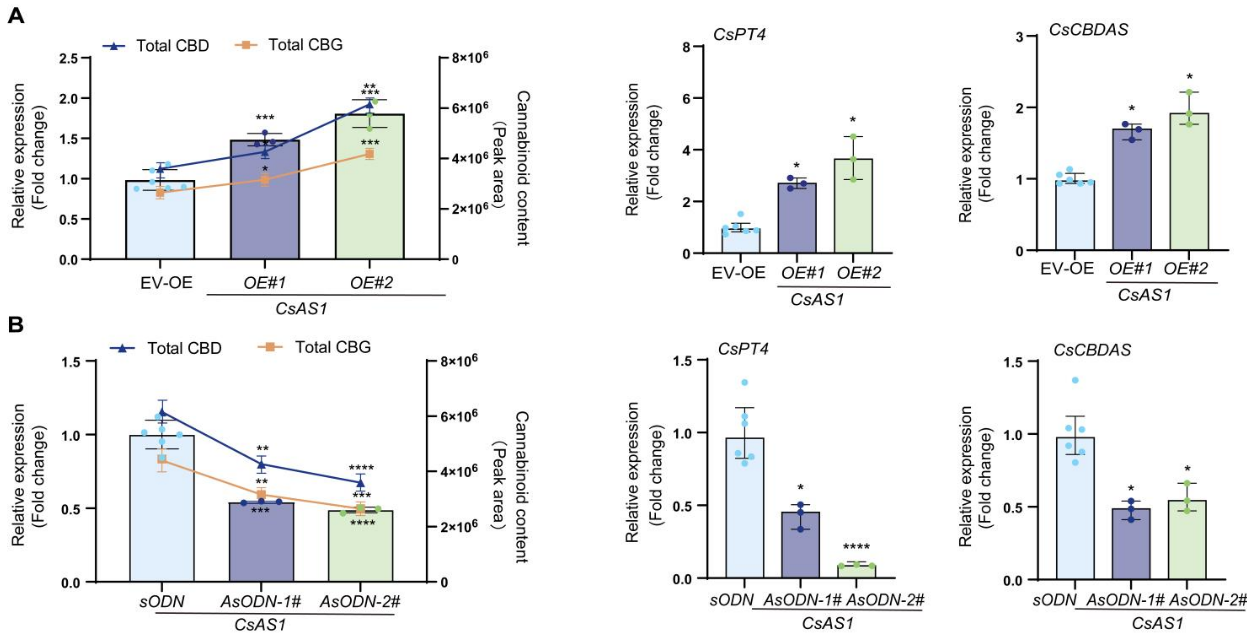


**Figure S10.** ***CsAS1* regulates the biosynthesis of the total CBD and CBG in *C. sativa.*** A) Total CBD and CBG accumulation and gene expression in cannabis leaves transiently overexpressing *CsAS1*. B) Total CBD and CBG content (left), and CsPT4 (middle) and CsCBDAS (right) expression in AsODN-mediated *CsAS1*-suppressed lines. Data represent means ± SD at least three independent experiments. Statistical significance was determined by Student's *t*-test compared to the corresponding control: ****$P < 0.0001$, ***$P < 0.001$, **$P < 0.01$, *$P < 0.05$, and ns, no significance.

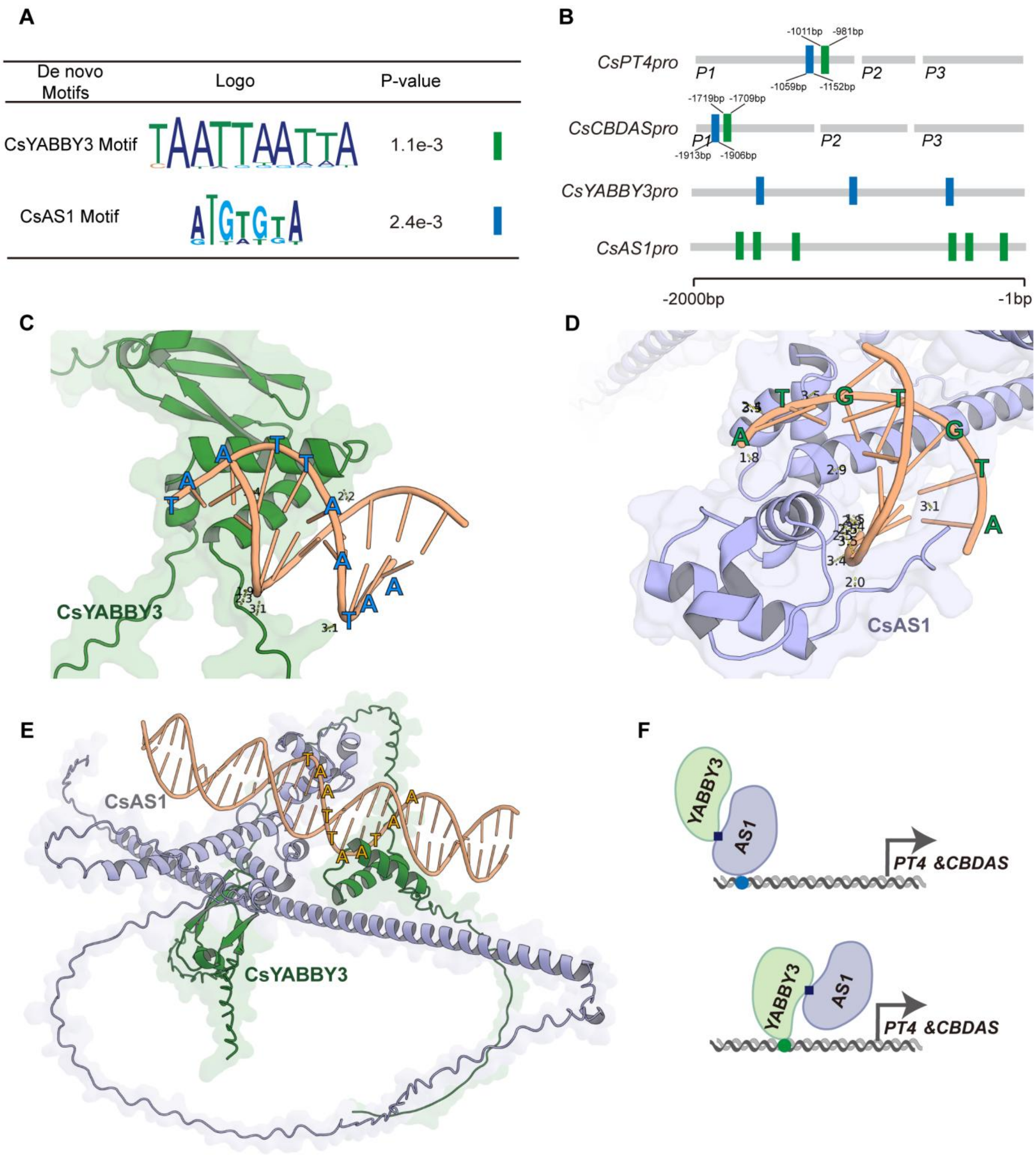


**Figure S11. Proposed DNA-binding modes and structural modeling of the CsYABBY3-CsAS1 regulatory complex.** A) The top-ranked de novo binding motifs enriched in the CsYABBY3 and CsAS1 DAP-seq data. B) Distribution of the identified consensus motifs for CsYABBY3 (blue boxes) and CsAS1 (green boxes) across the promoters of CsPT4, CsCBDAS, CsYABBY3, and CsAS1. C-D) Structural modeling of the CsYABBY3 (C) and CsAS1 (D) proteins interacting with their respective target DNA motifs. Key hydrogen bonds at the protein-DNA interfaces are highlighted. E) Molecular docking simulation of the CsYABBY3-CsAS1 heterodimer complex co-binding to a single CsYABBY3 binding site, suggesting that complex formation does not hinder, but potentially stabilizes, DNA binding. Structural modeling of CsAS1-motif interface. F) Proposed working models of CsYABBY3 and CsAS1 co-occupancy at target gene promoters. Given the spatial constraints between the two motifs, the TFs likely operate via a mutual recruitment mechanism. Either CsAS1 or CsYABBY3 can independently anchor to its respective motif (blue or green dots, respectively) and subsequently recruit its interacting partner to form a functional complex in situ. This complex formation acts to amplify the transcriptional activation of downstream targets such as *CsPT4* and *CsCBDAS*.
