# Supplementary material for: A Novel CsYABBY3‐CsAS1 Feedback Loop Coordinates Trichome Differentiation and Cannabinoid Biosynthesis in Cannabis sativa L
Source: Adv Sci (Weinh). 2026 Apr 2;13(34):e75055. doi: 10.1002/advs.75055 (PMC13285160; doi:10.1002/advs.75055)
Supplement: Supplementary file 2 — Supporting Table 1: advs75055‐sup‐0002‐Supplementary Table 1.pdf. [file ADVS-13-e75055-s004.pdf]

Supplementary Table 1 The summarized gene information for the phylogenetic classification of CsYABBYs

Name	Species	Gene ID
CsYABBY1	*Cannabis sativa*	Cs_C05H1G183830
CsYABBY2	*Cannabis sativa*	Cs_C07H1G284650
CsYABBY3	*Cannabis sativa*	Cs_C01H1G013660
CsYABBY4	*Cannabis sativa*	Cs_C09H1G358710
CsYABBY5	*Cannabis sativa*	Cs_C02H1G050890
AtYAB1	*Arabidopsis thaliana*	At2g45190
AtYAB2	*Arabidopsis thaliana*	At1g08465
AtYAB3	*Arabidopsis thaliana*	At4g00180
AtYAB5	*Arabidopsis thaliana*	At2g26580
AtCRC	*Arabidopsis thaliana*	At1g69180
AtINO	*Arabidopsis thaliana*	At1g23420
OsYAB5	*Oryza sativa*	Os04g0536300
OsYAB1	*Oryza sativa*	Os07g0160100
OsYAB3	*Oryza sativa*	Os10g0508300
OsYAB4	*Oryza sativa*	Os02g0643200
CmDRP	*Chrysanthemum morifolium*	UYL81895.1
LOC_Os04g45330	*Oryza sativa* 'Japonica Group'	LOC_Os04g45330
LOC_Os07g38410	*Oryza sativa* 'Japonica Group'	LOC_Os07g38410
LOC_Os07g06620	*Oryza sativa* 'Japonica Group'	LOC_Os07g06620
LOC_Os03g44710	*Oryza sativa* 'Japonica Group'	LOC_Os03g44710
LOC_Os03g11600	*Oryza sativa* 'Japonica Group'	LOC_Os03g11600
LOC_Os02g42950	*Oryza sativa* 'Japonica Group'	LOC_Os02g42950
LOC_Os12g42610	*Oryza sativa* 'Japonica Group'	LOC_Os12g42610
DCAR_031517	*Daucus carota*	DCAR_031517
DCAR_027801	*Daucus carota*	DCAR_027801
DCAR_026683	*Daucus carota*	DCAR_026683
DCAR_008464	*Daucus carota*	DCAR_008464
DCAR_007074	*Daucus carota*	DCAR_007074
DCAR_004921	*Daucus carota*	DCAR_004921
DCAR_008543	*Daucus carota*	DCAR_008543
DCAR_006190	*Daucus carota*	DCAR_006190
DCAR_012254	*Daucus carota*	DCAR_012254
DCAR_030050	*Daucus carota*	DCAR_030050
DCAR_014892	*Daucus carota*	DCAR_014892
Lsat_1_v5_gn_7_9041	*Lactuca sativa*	Lsat_1_v5_gn_7_9041
Lsat_1_v5_gn_7_43360	*Lactuca sativa*	Lsat_1_v5_gn_7_43360
Lsat_1_v5_gn_3_128981	*Lactuca sativa*	Lsat_1_v5_gn_3_128981
Lsat_1_v5_gn_3_53601	*Lactuca sativa*	Lsat_1_v5_gn_3_53601
Lsat_1_v5_gn_3_3040	*Lactuca sativa*	Lsat_1_v5_gn_3_3040
Lsat_1_v5_gn_0_27800	*Lactuca sativa*	Lsat_1_v5_gn_0_27800
Lsat_1_v5_gn_6_2400	*Lactuca sativa*	Lsat_1_v5_gn_6_2400
Lsat_1_v5_gn_8_58340	*Lactuca sativa*	Lsat_1_v5_gn_8_58340
Lsat_1_v5_gn_5_94941	*Lactuca sativa*	Lsat_1_v5_gn_5_94941
HanXRQChr10g0315651	*Smallanthus sonchifolius*	HanXRQChr10g0315651
HanXRQChr04g0122691	*Smallanthus sonchifolius*	HanXRQChr04g0122691
HanXRQChr05g0139121	*Smallanthus sonchifolius*	HanXRQChr05g0139121
HanXRQChr13g0423101	*Smallanthus sonchifolius*	HanXRQChr13g0423101
HanXRQChr13g0395021	*Smallanthus sonchifolius*	HanXRQChr13g0395021
HanXRQChr12g0375091	*Smallanthus sonchifolius*	HanXRQChr12g0375091
HanXRQChr12g0356171	*Smallanthus sonchifolius*	HanXRQChr12g0356171
HanXRQChr14g0446751	*Smallanthus sonchifolius*	HanXRQChr14g0446751
HanXRQChr17g0549371	*Smallanthus sonchifolius*	HanXRQChr17g0549371

HanXRQChr17g0539491	*Smallanthus sonchifolius*	HanXRQChr17g0539491
HanXRQChr06g0164191	*Smallanthus sonchifolius*	HanXRQChr06g0164191
HanXRQChr06g0169631	*Smallanthus sonchifolius*	HanXRQChr06g0169631
HanXRQChr03g0067451	*Smallanthus sonchifolius*	HanXRQChr03g0067451
HanXRQChr15g0476711	*Smallanthus sonchifolius*	HanXRQChr15g0476711
Glyma.04G094800	*Glycine max*	Glyma.04G094800
Glyma.17G138200	*Glycine max*	Glyma.17G138200
Glyma.17G113400	*Glycine max*	Glyma.17G113400
Glyma.02G121100	*Glycine max*	Glyma.02G121100
Glyma.12G096000	*Glycine max*	Glyma.12G096000
Glyma.12G190500	*Glycine max*	Glyma.12G190500
Glyma.08G285200	*Glycine max*	Glyma.08G285200
Glyma.01G063500	*Glycine max*	Glyma.01G063500
Glyma.01G029300	*Glycine max*	Glyma.01G029300
Glyma.01G137200	*Glycine max*	Glyma.01G137200
Glyma.13G157800	*Glycine max*	Glyma.13G157800
Glyma.13G311200	*Glycine max*	Glyma.13G311200
Glyma.05G056000	*Glycine max*	Glyma.05G056000
Glyma.06G308900	*Glycine max*	Glyma.06G308900
Glyma.06G096500	*Glycine max*	Glyma.06G096500
Glyma.03G029800	*Glycine max*	Glyma.03G029800
Glyma.18G140400	*Glycine max*	Glyma.18G140400
ZmB84.05G082000	*Zea may*	ZmB84.05G082000
ZmB84.05G299400	*Zea may*	ZmB84.05G299400
ZmB84.05G066800	*Zea may*	ZmB84.05G066800
ZmB84.07G213600	*Zea may*	ZmB84.07G213600
ZmB84.07G019400	*Zea may*	ZmB84.07G019400
ZmB84.01G069900	*Zea may*	ZmB84.01G069900
ZmB84.01G346000	*Zea may*	ZmB84.01G346000
ZmB84.01G416700	*Zea may*	ZmB84.01G416700
ZmB84.01G253400	*Zea may*	ZmB84.01G253400
ZmB84.03G128700	*Zea may*	ZmB84.03G128700
ZmB84.09G220900	*Zea may*	ZmB84.09G220900
ZmB84.10G186500	*Zea may*	ZmB84.10G186500
ZmB84.02G082700	*Zea may*	ZmB84.02G082700
Solyc07g008180	*Solanum lycopersicum*	Solyc07g008180
Solyc05g005240	*Solanum lycopersicum*	Solyc05g005240
Solyc05g012050	*Solanum lycopersicum*	Solyc05g012050
Solyc11g071810	*Solanum lycopersicum*	Solyc11g071810
Solyc08g079100	*Solanum lycopersicum*	Solyc08g079100
Solyc06g073920	*Solanum lycopersicum*	Solyc06g073920
Solyc01g091010	*Solanum lycopersicum*	Solyc01g091010
Solyc01g010240	*Solanum lycopersicum*	Solyc01g010240
Solyc12g009580	*Solanum lycopersicum*	Solyc12g009580
