# Supplementary material for: A Novel CsYABBY3‐CsAS1 Feedback Loop Coordinates Trichome Differentiation and Cannabinoid Biosynthesis in Cannabis sativa L
Source: Adv Sci (Weinh). 2026 Apr 2;13(34):e75055. doi: 10.1002/advs.75055 (PMC13285160; doi:10.1002/advs.75055)
Supplement: Supplementary file 3 — Supporting Table 2: advs75055‐sup‐0003‐Supplementary Table 2.pdf. [file ADVS-13-e75055-s002.pdf]

Supplementary Table 2 Information of yeast library.

Gene ID	Species	Name
Cs_C01H1G009910.1	*Cannabis sativa*	phosphoribulokinase, chloroplastic
Cs_C10H1G402630.2	*Cannabis sativa*	1,4-alpha-glucan-branching enzyme 1
Cs_C03H1G093920.1	*Cannabis sativa*	auxin-responsive protein IAA1
Cs_C09H1G365360.1	*Cannabis sativa*	AS1
Cs_C06H1G242930.2	*Cannabis sativa*	electron transfer flavoprotein subunit alpha
Cs_C05H1G188200.1	*Cannabis sativa*	beta-galactosidase
Cs_C03H1G082050.2	*Cannabis sativa*	L-ascorbate peroxidase, cytosolic
Cs_C03H1G093450.1	*Cannabis sativa*	low-specificity L-threonine aldolase 1
Cs_C10H1G395330.1	*Cannabis sativa*	large ribosomal subunit protein bL21c
Cs_C10H2G440000.1	*Cannabis sativa*	OXIDATIVE STRESS 3 LIKE 1
Cs_C07H2G291490.2	*Cannabis sativa*	naringenin,2-oxoglutarate 3-dioxygenase-like
Cs_C05H2G210570.1	*Cannabis sativa*	glyoxylate/hydroxypyruvate reductase HPR3
