# Supplementary material for: A Novel CsYABBY3‐CsAS1 Feedback Loop Coordinates Trichome Differentiation and Cannabinoid Biosynthesis in Cannabis sativa L
Source: Adv Sci (Weinh). 2026 Apr 2;13(34):e75055. doi: 10.1002/advs.75055 (PMC13285160; doi:10.1002/advs.75055)
Supplement: Supplementary file 4 — Supporting Table 3: advs75055‐sup‐0004‐Supplementary Table 3.pdf. [file ADVS-13-e75055-s001.pdf]

Supplementary Table 3 Primers used in this study.

Primer	Sequence (5'-3')	usage
pAbAi-CsPT4pro-motif 1-F	ATCATGAATGATTAGCAATATTGTCGACCTCGAGGCATGTGCT	
pAbAi-CsPT4pro-motif 1-R	AATATTGCTAATCATTCATGATAGATCCCCGGGTACCGAGCTCG	
pAbAi-CsPT4pro-mutant 1-F	ATCATGCCGACGGCGCAATATTGTCGACCTCGAGGCATGTGCT	
pAbAi-CsPT4pro-mutant 1-R	AATATTGCGCCGTCGGCATGATAGATCCCCGGGTACCGAGCTCG	
pAbAi-CsPT4pro-motif 2-F	ATTGTGTTAATTATGGCCCGAGTCGACCTCGAGGCATGTGCT	
pAbAi-CsPT4pro-motif 2-R	TCGGGCCATAATTAACACAATAGATCCCCGGGTACCGAGCTCG	
pAbAi-CsPT4pro-mutant 2-F	ATTGTGGGCCGGCGGGCCCGAGTCGACCTCGAGGCATGTGCT	
pAbAi-CsPT4pro-mutant 2-R	TCGGGCCCGCCGGCCCACAATAGATCCCCGGGTACCGAGCTCG	
pAbAi-CsPT4pro-motif 3-F	AATAGTATAATTATTTGAACGTCGACCTCGAGGCATGTGCT	
pAbAi-CsPT4pro-motif 3-R	GTTCAAATAATTATACTATTAGATCCCCGGGTACCGAGCTCG	Y1H
pAbAi-CsPT4pro-mutant 3-F	AATAGGCGCCGGCGTTGAACGTCGACCTCGAGGCATGTGCT	
pAbAi-CsPT4pro-mutant 3-R	GTTCAACGCCGGCGCCTATTAGATCCCCGGGTACCGAGCTCG	
pAbAi-CsPT4pro-motif 4-F	AATGGTTTATTATTTTTATTGTCGACCTCGAGGCATGTGCT	
pAbAi-CsPT4pro-motif 4-R	AATAAAAATAATAAACCATTAGATCCCCGGGTACCGAGCTCG	
pAbAi-CsPT4pro-mutant 4-F	AATGGGGGCGGCGGGTTTATTGTCGACCTCGAGGCATGTGCT	
pAbAi-CsPT4pro-mutant 4-R	AATAAACCCGCCGCCCCCATTAGATCCCCGGGTACCGAGCTCG	
pAbAi-CsPT4pro-motif 5-F	TTAATTAATAATTAAAATAGGTCGACCTCGAGGCATGTGCT	
pAbAi-CsPT4pro-motif 5-R	CTATTTTAATTATTAATTAAAGATCCCCGGGTACCGAGCTCG	
pAbAi-CsPT4pro-mutant 5-F	TTAATTCCGCCGGCAAATAGGTCGACCTCGAGGCATGTGCT	
pAbAi-CsPT4pro-mutant 5-R	CTATTTGCCGGCGGAATTAAAGATCCCCGGGTACCGAGCTCG	
pAbAi-CsYABBY3pro-kpn1-F	AAGCTTGAATTCGAGCTCGGTACCCCCTAATTATATATAGGATAGATACAC	
pAbAi-CsYABBY3pro-sal1-R	AGCACATGCCTCGAGGTCGACTTTTTTTTGGATTCGATTC	Y1H
pAbAi-CsAS1pro-kpn1-F	AAGCTTGAATTCGAGCTCGGTACCGTCCAAAATACCCTTATACACATAAC	
pAbAi-CsAS1pro-sal1-R	AGCACATGCCTCGAGGTCGACTTCATCACCATTTTCACGCT	
CsYABBY3-F-Ecor1-AD	GCCATGGAGGCCAGTGAATTCATGTCATCTTCTTCTGCTTC	
CsYABBY3-R-bamh1-AD	AGCTCGAGCTCGATGGATCCGTAAGGAGAAACACCAACTT	
CsYABBY3-P1-F-Ecor1-AD	GCCATGGAGGCCAGTGAATTCATGTCATCTTCTTCTGCTTC	
CsYABBY3-P1-R-bamh1-AD	AGCTCGAGCTCGATGGATCCACAAACTGCTGCAAGGAA	
CsYABBY3-P2-F-Ecor1-AD	GCCATGGAGGCCAGTGAATTCTCAAGAGTGTGACACTA	
CsYABBY3-P2-R-bamh1-AD	AGCTCGAGCTCGATGGATCCGATTATGGTGAGAAGCA	
CsYABBY3-P3-F-Ecor1-AD	GCCATGGAGGCCAGTGAATTCTCCTGGAAGAGATACCAAA	
CsYABBY3-P3-R-bamh1-AD	AGCTCGAGCTCGATGGATCCGTAAGGAGAAACACCAACTT	
AtYAB1-F-Ecor1-AD	gccatGGAGGCCAGTGAATTCATGTCTATGTCGTCTATGTC	
AtYAB1-R-bamh1-AD	AGCTCGAGCTCGATGGATCCATAAGGAGTCACACCAACGT	
AtYAB3-F-Ecor1-AD	gccatGGAGGCCAGTGAATTCATGTCGAGCATGTCCATGTC	
AtYAB3-R-bamh1-AD	AGCTCGAGCTCGATGGATCCGTTATGGGCCACCCCAACGTT	
AtAS1-F-Ecor1-AD	gccatGGAGGCCAGTGAATTCATGAAAGAGAGACAACGTT	
AtAS1-R-bamh1-AD	AGCTCGAGCTCGATGGATCCGGGCGGTCTAATCTGCAAC	
AtYAB3-mutant-F	TTGGACTCgcaGCTGACCATCCTCCCACGAAGAAGGCTAACGT	
AtYAB3-mutant-R	GGATGGTCAGCTGCGAGTCCAAAGTGTATGTGAGGGAAATGAG	
AtYAB3-mutant2-F	TTGGACTCGTGCCAGACCATCCTCCCACGAAGAAGGCTAACGT	
AtYAB3-mutant2-R	GGATGGTCTGGCACGAGTCCAAAGTGTATGTGAGGGAAATGAG	
GmFILa-F-Ecor1-AD	gccatGGAGGCCAGTGAATTCATGTCATCCTCTTCCAGCACAT	
GmFILa-R-bamh1-AD	AGCTCGAGCTCGATGGATCCGTAGGGTGAGACACCAACATTA	
SlYABBY1b-F-Ecor1-AD	gccatGGAGGCCAGTGAATTCATGTCCTCTTCAAATAGCTTAT	
SlYABBY1b-R-bamh1-AD	AGCTCGAGCTCGATGGATCCGTAAGGAGACACACTAACATT	
GmAS1-F-Ecor1-AD	gccatGGAGGCCAGTGAATTCATGAAGGATAGGCAACGTTG	
GmAS1-R-bamh1-AD	AGCTCGAGCTCGATGGATCCTCTTCCATTTGGTTCAGTGA	Y2H
Slphan-F-Ecor1-AD	gccatGGAGGCCAGTGAATTCATGAGGGAGAGGCAACGGT	

Slphan-R-bamh1-AD	AGCTCGAGCTCGATGGATCCGCGGCCGCCATTAGGTTCAGCAA	
CsYABBY3-F-Ecor1-BD	ATGGCCATGGAGGCCAGTGAATTCATGTCATCTTCTTCTGCTTC	
CsYABBY3-R-bamh1-BD	TGCAGCTCGAGCTCGATGGATCCCGTAAGGAGAAACACCAACTT	
CsYABBY3-P1-F-Ecor1-BD	ATGGCCATGGAGGCCAGTGAATTCATGTCATCTTCTTCTGCTTC	
CsYABBY3-P1-R-bamh1-BD	TGCAGCTCGAGCTCGATGGATCCACAAACTGCTGCAAGGAA	
CsYABBY3-P2-F-Ecor1-BD	ATGGCCATGGAGGCCAGTGAATTCTCAAGAGTGTGACACTA	
CsYABBY3-P2-R-bamh1-BD	TGCAGCTCGAGCTCGATGGATCCGATTATGGTGAGAAGCA	
CsYABBY3-P3-F-Ecor1-BD	ATGGCCATGGAGGCCAGTGAATTCTCCTGGAAGAGATACCAAA	
CsYABBY3-P3-R-bamh1-BD	TGCAGCTCGAGCTCGATGGATCCGTAAGGAGAAACACCAACTT	
CsAS1-F-Ecor1-BD	CATATGGCCATGGAGGCCGAATTC	
CsAS1-R-bamh1-BD	CGGCCGCTGCAGGTCGACGGATCC	
GmFILa-F-Ecor1-BD	CATATGGCCATGGAGGCCGAATTCATGTCATCCTCTTCCAGCACAT	
GmFILa-R-bamh1-BD	CGGCCGCTGCAGGTCGACGGATCCGTAGGGTGAGACACCAACATTA	
SlYABBY1b-F-Ecor1-BD	CATATGGCCATGGAGGCCGAATTCATGTCCTCTTCAAATAGCTTAT	
SlYABBY1b-R-bamh1-BD	CGGCCGCTGCAGGTCGACGGATCCGTAAGGAGACACACTAACATT	
GmAS1-F-Ecor1-BD	CATATGGCCATGGAGGCCGAATTCATGAAGGATAGGCAACGTTG	
GmAS1-R-bamh1-BD	CGGCCGCTGCAGGTCGACGGATCCTCTTCCATTTGGTTCAGTGA	
Slphan-F-Ecor1-BD	CATATGGCCATGGAGGCCGAATTCATGAGGGAGAGGCAACGGT	
Slphan-R-bamh1-BD	CGGCCGCTGCAGGTCGACGGATCCGCGGCCGCCATTAGGTTCAGCAA	
SlYABBY1b-mutant-F	TTGGTCTCGCACCTGATCAGACTGTTAAGAGGACTAATGTGC	
SlYABBY1b-mutant-R	GTCTGATCAGGTGCGAGACCAAATTGAATGTGTGGAAAGTGGG	
GmFILa-mutant-F	TTGGTCTCGCACCTGATCAGACTGTGAAGAAGACAAATGTGT	
GmFILa-mutant-R	GTCTGATCAGGtgcGAGACCAAAGTGGATGTGTGGGAAGTGG	
CsAS1-F-bamh1-cp096-infu	AACACGGGGGACTCTTGAGGATCCATGAAGGAGCGTCAGCGTTG	
CsAS1-R-ecor1-cp096-infu	TCCCCGGGTACCGAGCTCGAATTCATTTGGACGAGTACTAGTGTTG	Tomato transformation
CsYABBY3-F-bamh1-cp096-infu	AACACGGGGGACTCTTGAGGATCCATGTCATCTTCTTCTGCTTC	
CsYABBY3-R-ecor1-cp096-infu	TCCCCGGGTACCGAGCTCGAATTCGTAAGGAGAAACACCAACTT	
CsAS1-F-ruby	ATTTGGAGAGGACAGAATTCATGAAGGAGCGTCAGCGTTG	
CsAS1-R-ruby	GCCAAATGTTTGAACGATCTGCAGATTTGGACGAGTACTAGTGTTG	Over-expression
CsYABBY3-F-ruby	GAGAGGACAGAATTCGTCGACATGTCATCTTCTTCTGCTTC	
CsYABBY3-R-ruby	ATAAGGGTAGCTACCGTCGACGTAAGGAGAAACACCAACTT	
CsYABBY3-TRV2-ecor1-F	TGTGAGTAAGGTTACCGAATTCTATTGCCCCAGCAACTGG	
CsYABBY3-TRV2-CsAS1-R	ACGCTGACGCTCCTTCATTTGATTGATTCATCAGAA	
CsAS1-TRV2-CsYABBY3-F	TTCTGATGAATCAATCAAATGAAGGAGCGTCAGCGT	VIGS
CsAS1-TRV2-bamh1-R	CGTGAGCTCGGTACCGGATCCCCACCACTTTCCGAGTCTCT	
CsAS1-TRV2-ecor1-F	TGTGAGTAAGGTTACCGAATTCATGAAGGAGCGTCAGCGT	
CsAS1-TRV2-bamh1-R	CGTGAGCTCGGTACCGGATCCCCACCACTTTCCGAGTCTCT	
CsYABBY3-RNAi-F	TTGGAGAGGACACGCCATGGTATTGCCCCAGCAACTGG	
CsYABBY3-RNAi-R	CCTTACCAAGCTGGGGTACCTTGATTGATTCATCAGAA	
reCsYABBY3-RNAi-F	AAATCGATAAGCTTGGATCCTTGATTGATTCATCAGAA	
reCsYABBY3-RNAi-R	ACGTCGACCACGTGTCTAGATATTGCCCCAGCAACTGG	RNAi
CsCsAS1-RNAi-F	TTGGAGAGGACACGCCATGGATGAAGGAGCGTCAGCGT	
CsCsAS1-RNAi-R	CCTTACCAAGCTGGGGTACCCCACCACTTTCCGAGTCTCT	
reCsCsAS1-RNAi-F	AAATCGATAAGCTTGGATCCCCACCACTTTCCGAGTCTCT	
reCsCsAS1-RNAi-R	ACGTCGACCACGTGTCTAGAATGAAGGAGCGTCAGCGT	
CsYABBY3-AsODN-1	GCAGGGTTAAAGAAAGAGTG	
CsYABBY3-AsODN-2	TGAAACTGATTAGGCGAAGG	
CsYABBY3-AsODN-3	CGATTGTATGCCGAGGGAAC	
CsYABBY3-sODN-1	CACTCTTTCTTTAACCCTGC	
CsYABBY3-sODN-2	CCTTCGCCTAATCAGTTTCA	
CsYABBY3-sODN-3	GTTCCCTCGGCATACAATCG	

CsAS1-AsODN-1	TGGTCTCTGTACTCGTTCTC	AsODN
CsAS1-AsODN-2	GGTCGCGTTGGAGTGGCTTT	
CsAS1-AsODN-3	GGGGGTGTTTTTATAAGAAG	
CsAS1-sODN-1	GAGAACGAGTACAGAGACCA	
CsAS1-sODN-2	AAAGCCACTCCAACGCGACC	
CsAS1-sODN-3	CTTCTTATAAAAACACCCCC	
OAC-AsODN-1	TTTTGGGCTTCTGTGATTTC	
OAC-AsODN-2	GTACCCTTCTTCCTTATTCT	
OAC-AsODN-3	ATGTAGTCCTGAATAGTCTC	
OAC-sODN-1	GAAATCACAGAAGCCCAAAA	
OAC-sODN-2	AGAATAAGGAAGAAGGGTAC	
OAC-sODN-3	GAGACTATTCAGGACTACAT	
CsYABBY3-F-kpn1-coldTF	TAGGCATATGGAGCTCGGTACCATGTCATCTTCTTCTGCTTC	Prokaryotic expression
CsYABBY3-R-ecor1-coldTF	TGCAGGTCGACAAGCTTGAATTCGTAAGGAGAAACACCAACTT	
CsAS1-F-kpn1-coldTF	TAGGCATATGGAGCTCGGTACCATGAAGGAGCGTCAGCGTTG	
CsAS1-R-ecor1-coldTF	TGCAGGTCGACAAGCTTGAATTCATTTGGACGAGTACTAGTGTTG	
EF1a-F	ACCAAGATTGACAGGCGTTC	RT-qPCR
EF1a-R	CCTTCTTCTCCACAGCCTTG	
qCBDAS-F	CAATTCCAGAATCTGTATTTGTCC	
qCBDAS-R	TTCTTGCTTCTCCCAACTACATA	
qPT4-F	ATTCAGTTGGGGTTTGATGTGGAAG	
qPT4-R	AAGAGCTGATGTGGTTGCAGAAT	
qOAC-F	TGTTGGATTTGGAGATGTCTATCG	
qOAC-R	TTCGTGGTGTGTAGTCAAAAATGA	
qCsYABBY3-F	GAGATGTGGACACTGCACCA	
qCsYABBY3-R	CTCCCCTAGGTGGCACTACT	
qCsAS1-F	TCCTGCACTTCTTCGACACC	
qCsAS1-R	CTCAACCGACCTTCCTCCAC	
CsYABBY3-PNL-sac1-F	AGAACACGGGGGACGAGCTCATGTCATCTTCTTCTGCTTC	LCI
CsYABBY3-PNL-bamh1-R	GCCGGGCCCTCTAGAGGATCCGTAAGGAGAAACACCAACTT	
CsAS1-PCL-kpn1-F	TACGCGTCCCGGGGCGGTACCATGAAGGAGCGTCAGCGTTG	
CsAS1-PCL-sal1-R	ACGAAAGCTCTGCAGGTCGACATTTGGACGAGTACTAGTGTTG	
CsYABBY3pro-F	CCCTAATTATATATAGGATAGATACAC	Dual Luciferase
CsYABBY3pro-R	TTTTTTTTGGATTCGATTC	
CsYABBY3pro-0800-apa1-F	GCGAATTGGGTACCGGGCCCCCCTAATTATATATAGGATAGATACAC	
CsYABBY3pro-0800-bamh1-R	GGCCGCTCTAGAACTAGTGGATCCTTTTTTTTGGATTCGATTC	
CsAS1pro-F	GTCCAAAATACCCTTATACACATAAC	
CsAS1pro-R	TTCATCACCATTTTCACGCT	
CsAS1pro-0800-apa1-F	GCGAATTGGGTACCGGGCCCGTCCAAAATACCCTTATACACATAAC	
CsAS1pro-0800-bamh1-R	GGCCGCTCTAGAACTAGTGGATCCTTCATCACCATTTTCACGCT	
CsYABBY3-F-agl1-GFP-infu	CTGCCCAAATTCGCGACCGGTATGTCATCTTCTTCTGCTTC	Subcellular localization
CsYABBY3-R-agl1-GFP-infu	GCCCTTGCTCACCATACCGGTCGTAAGGAGAAACACCAACTT	
CsAS1-F-agl1-GFP-infu	CTGCCCAAATTCGCGACCGGTATGAAGGAGCGTCAGCGTTG	
CsAS1-R-agl1-GFP-infu	GCCCTTGCTCACCATACCGGTATTTGGACGAGTACTAGTGTTG	
CsYABBY3-BD-mutant#1-F	ATGTCAGCATCTGCAGCTTCAACAATATTGCCCCAGC	
CsYABBY3-BD-mutant#1-R	GAAGCTGCAGATGCTGACATGAATTCGGCCTCCATGGCCATATG	
CsYABBY3-BD-mutant#2-F	GGACACGCAACCAATCTGCTTCCGGTGAACATGTGT	
CsYABBY3-BD-mutant#2-R	AGCAGATTGGTTGCGTGTCCACATCTCACAGTTAGTGTC	
CsYABBY3-BD-mutant#3-F	GCATTGGGCGCATCTTTCTTTAACCCTGCTTCTCACCATA	
CsYABBY3-BD-mutant#3-R	AAGAAAGATGCGCCCAATGCAAACTGATTAGGCGAAGGCA	
CsYABBY3-BD-mutant#4-F	AAGAGACAGGCAGTTCCCTCGGCATACAATCGTTTCATCAAGG	

CsYABBY3-BD-mutant#4-R	GAGGGAACTGCCTGTCTCTTCTCTGGGGGTCTGTTAATAA	Y2H
CsYABBY3-BD-mutant#5-F	AGCATCAGCCGCAGCATTTCTGATGAATCAATCAA	
CsYABBY3-BD-mutant#5-R	ATGCTGCGGCTGATGCTGGTATCTCTTCCAGGA	
CsYABBY3-BD-mutant#6-F	AATGCATCAAGCATGGCTGATTTTGCAGTAGT	
CsYABBY3-BD-mutant#6-R	CCATGCTTGATGCATTCATCAGAAAGTTTGGGGC	
CsYABBY3-BD-mutant#7-F	TTTGGTCTCGCACCTGATCAGACTGGGAAGAAGACAA	
CsYABBY3-BD-mutant#7-R	ATCAGGTGCGAGACCAAAGTGAATGTGTGGAAAGTG	
CsYABBY3-BD-mutant#8-F	TTTTGCATCAGCCAATGCAGTTGGTGTTTCTCCTTAC	
CsYABBY3-BD-mutant#8-R	TGCATTGGCTGATGCAAAAAACCCATCCTTCATC	
CsYABBY3-1F	TAATACGACTCACTATAGGGCTGGACCACCTCTCTTCCTCA	In Situ Hybridization
CsYABBY3-2R	TAATACGACTCACTATAGGGCTACTGCAAAATCAGCCATGC	
