# Supplementary material for: A Novel CsYABBY3‐CsAS1 Feedback Loop Coordinates Trichome Differentiation and Cannabinoid Biosynthesis in Cannabis sativa L
Source: Adv Sci (Weinh). 2026 Apr 2;13(34):e75055. doi: 10.1002/advs.75055 (PMC13285160; doi:10.1002/advs.75055)
Supplement: Supplementary file 5 — Supporting Table 4: advs75055‐sup‐0005‐Supplementary Table 4.pdf. [file ADVS-13-e75055-s003.pdf]

Supplementary Table 4 Phylogenetic analysis of the FIL/YAB3 subclade of YABBY transcriptional factors

| **Classification** | **Order** | **Family** | **Species** | **Gene ID** |
|---|---|---|---|---|
| ANA | Amborellales | Amborellaceae | Amborella trichopoda | AmtrKAL4198622.1, AmtrKAL4195662.1, AmtrKAL4200441.1, AmtrKAL4198755.1 , AmtrKAL4184683.1 |
| ANA | Nymphaeales | Nymphaeaceae | Nymphaea colorata | Nycol.N00117.1, Nycol.A00487.1, Nycol.D01480.1, Nycol.B01098.1, Nycol.B00216.1, Nycol.G00354.1, Nycol.L00815.1 |
| ANA | Austrobaileyales | Schisandraceae | Schisandra chinensis | g70287.t1, g59969.t1, g77207.t1, g17306.t1, g20613.t1 |
| Magnoliids | Piperales | Aristolochiaceae | Aristolochia fimbriata | Af01G026600.1, Af02G072800.1, Af03G134500.1, Af05G185300.1, Af07G024800.1 |
| Magnoliids | Magnoliales | Annonaceae | Annona cherimola | Anche102Chr2g0051300.1, Anche102Chr2g0051650.1, Anche102Chr3g0055340.1, Anche102Chr3g0055730.1, Anche102Chr5g0038080.1, Anche102Chr5g0038100.1, Anche102Scf0005g0001760.1, Anche102Scf0009g0001540.1 |
| Monocots | Alismatales | Potamogetonaceae | Zostera marina | Zosma02g02190, Zosma01g01080, Zosma06g30530, Zosma06g02980, Zosma05g33270 |
| Monocots | Dioscoreales | Dioscoreaceae | Dioscorea alata | Dioal.16G035200.1.p, Dioal.15G078500.1.p, Dioal.13G012000.1.p, Dioal.05G148400.1.p, Dioal.02G015400.1.p, Dioal.08G126600.1.p |
| Monocots | Asparagales | Orchidaceae | Apostasia shenzhenica | ASH_rna10217, ASH_rna17651, ASH_rna18886, ASH_rna3490 |
| Monocots | Arecales | Arecaceae | Phoenix dactylifera | XP_008778120.1, XP_008785629.1, XP_008787013.1, XP_008790360.1, XP_008810626.2, XP_008810627.2, XP_008812180.3, XP_017698332.1, XP_038983486.1, XP_038983487.1 |
| Monocots | Poales | Gramineae | Oryza sativa | LOC_Os10g36420.1, LOC_Os12g42610.1, LOC_Os02g42950.1, LOC_Os03g11600.2, LOC_Os04g45330.1, LOC_Os07g06620.2, LOC_Os07g38410.1 |
| Monocots | Poales | Gramineae | Zea mays | Zm00001d002829_T001, Zm00001d013895_T002, Zm00001d018829_T001, Zm00001d021863_T001, Zm00001d025944_T001, Zm00001d028216_T001, Zm00001d031109_T002, Zm00001d032502_T001, Zm00001d033508_T001, Zm00001d041277_T002, Zm00001d048083_T001 |
| Eudicots | Ranunculales | Ranunculaceae | Coptis chinensis | Cch00028704-PA, Cch00023982-PA, Cch00033443-PA, Cch00002121-PA, Cch00003736-PA |
| Eudicots | Ranunculales | Papaveraceae | Papaver somniferum | PS1015680.1, PS0206010.1, PS0206020.1, PS0206030.1, PS0412300.1, PS0414520.1, PS0511010.1, PS0513290.1, PS0539180.1, PS0710750.1, PS0819560.1, PSUN43240.PSUN43240.1 |
| Rosids Core Eudic ots | Vitales | Vitaceae | Vitis vinifera | Vitvi01g00013_P001, Vitvi06g00972_P001, Vitvi11g00492_P001, Vitvi08g00274_P001,Vitvi15g00708_P001, Vitvi01g00703_P001, Vitvi02g00510_P001 |
| Rosids | Sapindales | Rutaceae | Citrus clementina | Ciclev10009560m, Ciclev10021834m, Ciclev10022170m, Ciclev10029354m, Ciclev10033714m, Ciclev10033770m |
| Rosids | Brassicales | Brassicaceae | Arabidopsis thaliana | AT1G08465.1, AT1G23420.2, AT1G69180.1, AT2G26580.1, AT2G45190.1 |
| Rosids | Rosales | Cannabaceae | Cannabis sativa | Cs_C05H1G183830, Cs_C07H1G284650, Cs_C09H1G358710, Cs_C01H1G013660, Cs_C02H1G050890 |
| Rosids | Rosales | Moraceae | Morus notabilis | MonoXP_010093672.1, MonoXP_010088882.1, MonoXP_024017521.1, MonoXP_010090516.1, MonoXP_010105229.1, MonoXP_024024007.1, MonoXP_024017519.1, MonoXP_024017520.1 |
| Rosids | Malpighiales | Euphorbiaceae | Ricinus communis | XM_002511626.3, XM_048370469.1, XM_048379966.1, XM_048379962.1, XM_002527155.4, XM_015727481.3, XM_048376377.1, XM_015723143.3, XM_048376379.1, XM_025158496.2, XM_015717278.3, XM_002515834.4 |

| | | | | |
|---|---|---|---|---|
| Rosids | Fabales | Fabaceae | Abrus precatorius | rna-XM_027485731.1, rna-XM_027485734.1, rna-XM_027485730.1, rna-XM_027485738.1, rna-XM_027485736.1, rna-XM_027485732.1, rna-XM_027485733.1, rna-XM_027485735.1, rna-XM_027485737.1, rna-XM_027490417.1, rna-XM_027490416.1, rna-XM_027492268.1, rna-XM_027506970.1, rna-XM_027505779.1, rna-XM_027476304.1, rna-XM_027480082.1, rna-XM_027479274.1, rna-XM_027479273.1 |
| Rosids | Cucurbitales | Cucurbitaceae | Lagenaria siceraria | Lsi07G013310.1, Lsi11G003260.1, Lsi04G005610.1, Lsi06G009810.1, Lsi09G003970.1, Lsi08G016690.1 |
| Rosids | Saxifragales | Crassulaceae | Rhodiola crenulata | CCG018987.1, CCG020078.1, CCG011262.1, CCG028060.1, CCG000956.1, CCG022305.1, CCG017250.2 |
| Superasterids | Caryophyllales | Polygonaceae | Fagopyrum tataricum | FtPinG0003162600.01.T01, FtPinG0000155700.01.T01, FtPinG0000507400.01.T01, FtPinG0000273100.01.T01, FtPinG0000211900.01.T01, FtPinG0003638300.01.T01, FtPinG0001121900.01.T01 |
| Asterids | Cornales | Nyssaceae | Camptotheca acuminata | Cac_g004636.t1, Cac_g010164.t1, Cac_g028617.t1, Cac_g032185.t1, Cac_g001678.t1, Cac_g005011.t1 |
| Asterids | Garryales | Eucommiaceae | Eucommia ulmoides | IMPTEUL1N79075_1, IMPTEUL1N62868_1, IMPTEUL1N65848_1, IMPTEUL1N17831_1, IMPTEUL1N117376_1, IMPTEUL1N85381_1 |
| Asterids | Gentianales | Apocynaceae | Catharanthus roseus | CrChr1.2999, CrChr2.2199, CrChr5.1213, CrChr6.3293, CrChr8.250 |
| Asterids | Lamiales | Lamiaceae | Salvia miltiorrhiza | Sm07A57T62.1, Sm05A9T87.1, Sm05G542T19.1, Sm08A218T35.1, Sm06A299T33.1 |
| Asterids | Solanales | Solanaceae | Solanum lycopersicum | Solyc06g073920.3.1, Solyc05g005240.2.1, Solyc05g012050.3.1, Solyc11g071810.2.1, Solyc12g009580.2.1, Solyc01g091010.3.1, Solyc01g010240.3.1, Solyc08g079100.3.1 |
| Asterids | Asterales | Campanulaceae | Platycodon grandiflorus | IMPTPGR2N29515_1, IMPTPGR2N23408_1, IMPTPGR2N8535_1, IMPTPGR2N81453_1, IMPTPGR2N45768_1 |
| Asterids | Asterales | Asteraceae | Lactuca sativa | Lsat_1_v5_gn_7_9041.1, Lsat_1_v5_gn_7_43360.1, Lsat_1_v5_gn_3_128981.1, Lsat_1_v5_gn_3_53601.1, Lsat_1_v5_gn_3_3040.1, Lsat_1_v5_gn_0_27800.1, Lsat_1_v5_gn_6_2400.1, Lsat_1_v5_gn_8_58340.3, Lsat_1_v5_gn_5_94941.2 |
| Asterids | Dipsacales | Caprifoliaceae | Lonicera japonica | Lj9A567T72.1, Lj6A777G36.1, Lj2C211G2.1 |
| Asterids | Apiales | Apiaceae | Daucus carota | DCAR_004921, DCAR_007074, DCAR_008464, DCAR_008543, DCAR_012254, DCAR_014892, DCAR_026683, DCAR_027801, DCAR_030050, DCAR_031517 |
